# Crystal structures and identification of novel Cd^2+^-specific DNA aptamer

**DOI:** 10.1093/nar/gkad239

**Published:** 2023-04-04

**Authors:** Hehua Liu, Yanqing Gao, Johnsi Mathivanan, Zev Armour-Garb, Zhiwei Shao, Yixi Zhang, Xin Zhao, Qiyuan Shao, Weizhen Zhang, Jie Yang, Chulei Cao, Huili Li, Jia Sheng, Jianhua Gan

**Affiliations:** Shanghai Public Health Clinical Center, State Key Laboratory of Genetic Engineering, Collaborative Innovation Center of Genetics and Development, Department of Biochemistry and Biophysics, School of Life Sciences, Fudan University, Shanghai 200438, China; Shanghai Public Health Clinical Center, State Key Laboratory of Genetic Engineering, Collaborative Innovation Center of Genetics and Development, Department of Biochemistry and Biophysics, School of Life Sciences, Fudan University, Shanghai 200438, China; Department of Chemistry and The RNA Institute, University at Albany, State University of New York, Albany, NY 12222, USA; Department of Chemistry and The RNA Institute, University at Albany, State University of New York, Albany, NY 12222, USA; Shanghai Public Health Clinical Center, State Key Laboratory of Genetic Engineering, Collaborative Innovation Center of Genetics and Development, Department of Biochemistry and Biophysics, School of Life Sciences, Fudan University, Shanghai 200438, China; Shanghai Public Health Clinical Center, State Key Laboratory of Genetic Engineering, Collaborative Innovation Center of Genetics and Development, Department of Biochemistry and Biophysics, School of Life Sciences, Fudan University, Shanghai 200438, China; Shanghai Public Health Clinical Center, State Key Laboratory of Genetic Engineering, Collaborative Innovation Center of Genetics and Development, Department of Biochemistry and Biophysics, School of Life Sciences, Fudan University, Shanghai 200438, China; Shanghai Public Health Clinical Center, State Key Laboratory of Genetic Engineering, Collaborative Innovation Center of Genetics and Development, Department of Biochemistry and Biophysics, School of Life Sciences, Fudan University, Shanghai 200438, China; Shanghai Public Health Clinical Center, State Key Laboratory of Genetic Engineering, Collaborative Innovation Center of Genetics and Development, Department of Biochemistry and Biophysics, School of Life Sciences, Fudan University, Shanghai 200438, China; Shanghai Public Health Clinical Center, State Key Laboratory of Genetic Engineering, Collaborative Innovation Center of Genetics and Development, Department of Biochemistry and Biophysics, School of Life Sciences, Fudan University, Shanghai 200438, China; Shanghai Public Health Clinical Center, State Key Laboratory of Genetic Engineering, Collaborative Innovation Center of Genetics and Development, Department of Biochemistry and Biophysics, School of Life Sciences, Fudan University, Shanghai 200438, China; Shanghai Public Health Clinical Center, State Key Laboratory of Genetic Engineering, Collaborative Innovation Center of Genetics and Development, Department of Biochemistry and Biophysics, School of Life Sciences, Fudan University, Shanghai 200438, China; Department of Chemistry and The RNA Institute, University at Albany, State University of New York, Albany, NY 12222, USA; Shanghai Public Health Clinical Center, State Key Laboratory of Genetic Engineering, Collaborative Innovation Center of Genetics and Development, Department of Biochemistry and Biophysics, School of Life Sciences, Fudan University, Shanghai 200438, China

## Abstract

Cadmium (Cd) is one of the most toxic heavy metals. Exposure to Cd can impair the functions of the kidney, respiratory system, reproductive system and skeletal system. Cd^2+^-binding aptamers have been extensively utilized in the development of Cd^2+^-detecting devices; however, the underlying mechanisms remain elusive. This study reports four Cd^2+^-bound DNA aptamer structures, representing the only Cd^2+^-specific aptamer structures available to date. In all the structures, the Cd^2+^-binding loop (CBL-loop) adopts a compact, double-twisted conformation and the Cd^2+^ ion is mainly coordinated with the G9, C12 and G16 nucleotides. Moreover, T11 and A15 within the CBL-loop form one regular Watson–Crick pair and stabilize the conformation of G9. The conformation of G16 is stabilized by the G8–C18 pair of the stem. By folding and/or stabilizing the CBL-loop, the other four nucleotides of the CBL-loop also play important roles in Cd^2+^ binding. Similarly to the native sequence, crystal structures, circular dichroism spectrum and isothermal titration calorimetry analysis confirm that several variants of the aptamer can recognize Cd^2+^. This study not only reveals the underlying basis for the binding of Cd^2+^ ions with the aptamer, but also extends the sequence for the construction of novel metal–DNA complex.

## INTRODUCTION

Cadmium (Cd) is one of the most toxic heavy metals known to date (1–4). It can induce abnormal production of reactive oxygen species (5,6), which can attack and damage the normal structures of proteins, DNAs and lipids in the cells. Cd can mimic zinc and calcium, and thus interferes with the metabolism of phosphorus and calcium (7). When Cd gets accumulated in the human body, it impairs the function of a variety of organs and systems such as the kidney (8–10), respiratory system (11–13), reproductive system (14,15) and skeletal system (16,17). In addition to the most serious itai–itai disease (7,18), Cd poisoning has also been linked with abortion (19,20), renal tubular dysfunction, cancers and many other diseases.

Cd contamination can be caused by both natural and anthropogenic activities. The natural sources of Cd pollution include volcanic activity (21), rock erosion and forest fires. The major Cd contaminations are caused by human activities, especially when Cd is used as an anticorrosive reagent in industries, as a stabilizer in polyvinyl chloride products, as a color pigment (22,23) and in the fabrication of Cd-containing batteries (24,25). As estimated, 70% of Cd used in the metal mining and refining industries and the Cd-containing battery production is scattered in the environment. Other human activities, such as non-ferrous metal smelters (26), recycling of electronic waste (27), fossil fuel combustion and use of phosphate fertilizers (28,29), can also release Cd into the environment.

Cd can be absorbed via the respiratory system, the gastrointestinal system and the dermal system of humans. Owing to the threats to human organs and systems, Cd containment has become a significant global concern. To detect and prevent the environmental Cd from entering the human body, several analytical methods (30), including inductively coupled plasma mass spectrometry (31), inductively coupled plasma atomic emission spectrometry (32) and atomic absorption spectrometry (33), have been developed. Although these methods are accurate, they require sophisticated equipment, which is neither suitable for on-site detection nor available in many developing countries. The requirement of complex sample treatment (34) is another major limitation of these methods, leading to high cost and long analysis time.

For the development of more rapid and simpler Cd detection methods, many different materials such as organic polymers, metal–organic frameworks, gold nanoparticles and nucleic acids have been investigated to date. It was confirmed that some DNA sequences possess high Cd ion (Cd^2+^) binding affinity and specificity. These sequences were termed Cd^2+^-specific aptamers and have been extensively utilized in the development of Cd^2+^ detectors (35–40). Compared to the traditional Cd^2+^-detecting methods, these aptamer-based methods are easier in sample handling and more cost effective. In addition to Cd^2+^, DNA aptamers have also been widely used in the detection of other heavy metal ions, such as Hg^2+^, Pb^2+^ and Ag^+^ (41–43). The structures of many DNA aptamers complexed with metals such as Hg^2+^, Pb^2+^ and Ag^+^ have been reported (44–50), which reveal the detailed mechanistic explanation on the metal–aptamer interactions. However, due to the lack of Cd^2+^-bound aptamer structure, how Cd^2+^ is recognized by the aptamer remains elusive. This study reports four crystal structures, circular dichroism (CD) spectra and isothermal titration calorimetry (ITC) analysis of a Cd^2+^-specific aptamer. All the structures show that Cd^2+^ is recognized by the Cd^2+^-binding loop (CBL-loop) and coordinates with the nucleobases of G9, C12 and G16. Other nucleotides of the loop and the G8–C18 pair of the stem also affect the Cd^2+^ binding, via proper folding and/or stabilizing the conformation of the CBL-loop. Overall, this study provides the structural basis for the rational development of Cd^2+^-specific detectors and functional DNA nanodevices.

## MATERIALS AND METHODS

### Chemicals

All chemicals and buffers used in this study were of analytical reagent grade. Sodium chloride, potassium chloride, magnesium chloride, Tris acetate and acetic acid were purchased from Sigma–Aldrich. Spermine tetrahydrochloride, sodium cacodylate, (+/−)-2-methyl-2,4-pentanediol (MPD) and cadmium chloride were purchased from Hampton Research.

### Crystallization and data collection

All DNAs utilized in the crystallization studies were purchased from the Shanghai GENERAY Biotech Co., Ltd, and dissolved in double-distilled water (ddH_2_O). The DNA sequences are listed in Supplementary Table S1. The crystallization samples were prepared at room temperature by mixing DNA and CdCl_2_, which was also dissolved in ddH_2_O. The final concentrations of the DNA and CdCl_2_ are summarized in Supplementary Table S2. Crystallizations were performed at 18°C by the hanging-drop vapor diffusion method. The droplet consisted of an equal volume of DNA sample and crystallization solution for all the complexes. The reservoir solution was composed of 30% (v/v) MPD for the native DNA1–Cd^2+^, the T10A–Cd^2+^ and the T22C–Cd^2+^ complexes, whereas identical crystallization solution was utilized in the drop and the reservoir for the C11G15–Cd^2+^ complex. The detailed compositions of the crystallization solution are also summarized in Supplementary Table S2. The DNA1–Cd^2+^ and the G22C–Cd^2+^ crystals grew within 2 days and reached their full sizes within 1 week. Growth of the C11G15–Cd^2+^ and the T10A–Cd^2+^ crystals was slow; it took ∼2 weeks for the crystals to form and another week to reach their full sizes.

All the crystals were cryoprotected using 30% (v/v) MPD and flash-frozen by quickly dipping into liquid nitrogen. The X-ray diffraction data were collected on beamline BL17U1 at Shanghai Synchrotron Radiation Facility and beamlines BL18U1 and BL19U1 at National Facility for Protein Science at cryogenic temperature. One single crystal was used for each structure, and data processing was carried out by using the HKL2000 or HKL3000 programs (51). The data collection and processing statistics are presented in Table 1.

**Table 1.** Data collection and refinement statistics

Structure	DNA1–Cd^2+^	T10A–Cd^2+^	C11G15–Cd^2+^	T22C–Cd^2+^
PDB ID	8GZJ	8GZK	8GZL	8GZM
Data collection^a^				
Space group	*C*2	*C*2	*P*2_1_	*P*4_3_2_1_2
Cell parameters				
* a* (Å)	81.5	28.7	26.8.	46.4
* b* (Å)	41.9	136.4	45.7	46.4
* c* (Å)	73.2	51.1	147.7	107.3
* α* (°)	90.0	90.0	90.0	90.0
* β* (°)	106.2	104.4	92.9	90.0
* γ* (°)	90.0	90.0	90.0	90.0
Wavelength (Å)	0.9793	0.9793	0.9793	0.9793
Resolution (Å)	30.0–2.10	30.0–2.95	29.4–3.0	30.0–2.55
High-resolution shell (Å)	2.18–2.10	3.06–2.95	3.18–3.0	2.64–2.55
Completeness (%)	93.5 (80.4)	97.4 (91.4)	84.3 (81.7)	99.1 (100.0)
Redundancy	6.0 (4.0)	5.1 (3.4)	4.9 (4.4)	22.4 (21.2)
* R*_merge_ (%)	6.1 (66.0)	9.1 (41.5)	9.8 (40.7)	7.7 (66.8)
* I*/*σ*(*I*)	22.6 (2.0)	17.8 (2.5)	7.3 (1.8)	28.2 (1.6)
Refinement				
Resolution (Å)	23.4–2.10	28.1–2.95	29.7–3.0	22.7–2.55
No. of reflections	10075	3634	5953	3810
* R*_work_ (%)**/***R*_free_ (%)	23.2/27.4	23.3/28.9	23.3/28.4	19.4/20.5
No. of atoms				
DNA	1467	1064	2043	510
Cd^2+^	3	2	5	2
Rms deviations				
Bond length (Å)	0.011	0.013	0.005	0.012
Bond angle (°)	1.166	1.384	0.825	1.437

^a^Values in parentheses are for the high-resolution shell.

### Structure determination and refinement

The structure of the native DNA1–Cd^2+^ was solved by the single anomalous diffraction method (52) by using the AutoSol program (53). Based on the initial electron density maps, the DNA models were manually built by using the graphic program Coot (54). Then, the DNA models were refined against the diffraction data by using the Refmac5 program (55) embedded in the CCP4i suite (56). Cd^2+^ ions were also manually built by using Coot. The C11G15–Cd^2+^, the T10A–Cd^2+^ and the G22C–Cd^2+^ complex structures were all solved by molecular replacement method by using the native DNA1–Cd^2+^ structure as the searching model. All final refinement of the structures was carried out by using the phenix.refine program embedded in the Phenix suite (57). The refinement statistics are summarized in Table 1. All structural analyses and presentations were performed by using the PyMol program.

### Circular dichroism experiments

All DNAs used in CD analysis were purchased from Integrated DNA Technologies. DNA samples were initially dissolved in RNase-free water, and the concentration was determined using nanodrop (absorbance at 260 nm). Cadmium nitrate tetrahydrate salt was used to prepare the Cd^2+^ ion stock solution. Tris–HAc (20 mM, pH 7.4), NaCl (140 mM), KCl (5 mM) and MgCl_2_ (10 mM) buffer was used to prepare CD samples.

CD experiments were performed at room temperature using a CD spectrometer (Jasco CD, J-815). Each sample was prepared by heating the mixture of DNA (10 μM) and Cd^2+^ ion (100 μM) at 95°C for 5 min and then slowly cooled down to room temperature. The CD spectra were obtained in the presence and absence of Cd^2+^ ions for all strands, respectively. The samples were scanned from 350 to 200 nm in a 1-mm quartz cuvette (Hellma Analytics) with a 1-nm data pitch. Three accumulations were performed for each sample with 1.0 nm bandwidth and 1.0 s digital integration time. CD spectra were recorded, and all the curves were plotted by using Sigma Plot (version 12.0). All CD spectra were baseline-corrected against the blank buffer.

### Isothermal titration calorimetry assays

All ITC experiments were performed on a MicroCal PEAQ-ITC calorimeter (MicroCal Inc.). Interaction was performed in a buffer composed of HEPES (10 mM, pH 7.4) and NaCl (100 mM) at 25°C. CdCl_2_ (500 μM) was titrated into the cell containing the native DNA1 (200 μl) or variants (40 μM). A total of 18 injections (each of 2 μl) were performed. The heat evolved following each titration point was obtained from the integral of the signal, and the data were analyzed by using MicroCal PEAQ-ITC analysis software.

### Analytical ultracentrifugation

Sedimentation velocity experiments were carried out by an OptimaAUC (Beckman Coulter, USA). A volume of 380 μl of annealed DNA1 (*A*_260_ = 0.75, 3.2 μM) with CdCl_2_ (32 μM) and 400 μl of matching buffer (10 mM HEPES, 100 mM NaCl, pH 7.4) were injected into appropriate channels of 12-mm double sector aluminum centerpieces. Solutions were centrifuged at 60 000 rpm at 20°C in an An-60Ti rotor for 8 h. Scans were collected at 260 nm, with 1 min elapsed between each scan. Data were analyzed by using the continuous sedimentation coefficient distribution *c*(*s*) model in SEDFIT software.

## RESULTS AND DISCUSSION

### High-resolution crystal structure of Cd^2+^-bound aptamer

**Figure 1.**
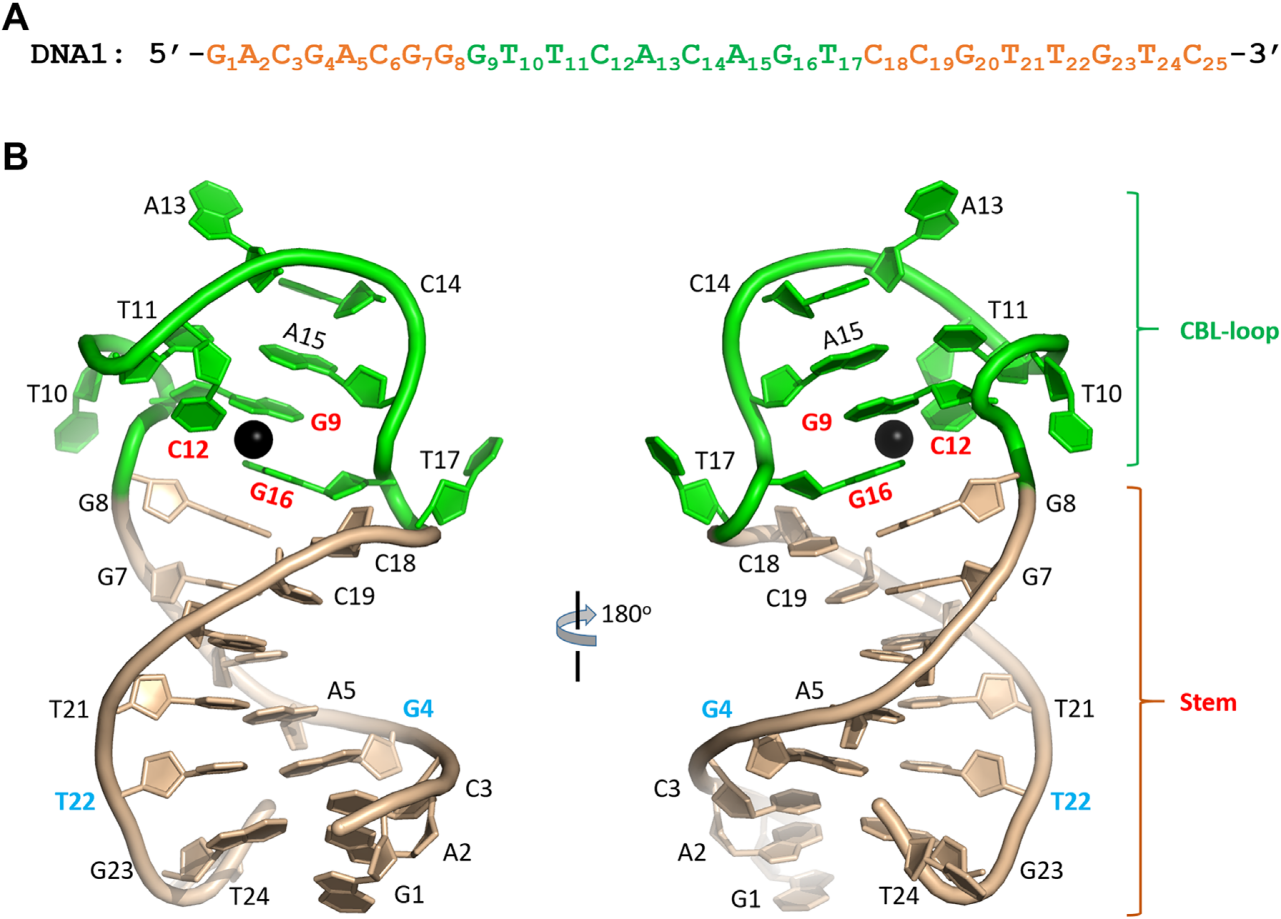
Crystal structure of the native DNA1–Cd^2+^ complex. (**A**) The sequence of the native DNA1 aptamer. (**B**) Cartoon-and-stick view presentation of the DNA1–Cd^2+^ complex, based on complex A in the asymmetric unit. The stem and the CBL-loop of DNA1 are colored in wheat and green, respectively. The bound Cd^2+^ ion is shown as sphere in black.

To reveal the underlying basis for Cd^2+^ binding by Cd^2+^-specific aptamers, herein, various DNA sequences were synthesized and extensive crystallization trials were performed. Although no crystal grew for other sequences, rod-like crystals were obtained for one DNA sequence (referred to as DNA1 hereinafter) in complex with Cd^2+^ ion. The sequence of DNA1 (Figure 1A) was derived from the Cd-2-1 aptamer discovered by Wang and coworkers (35). DNA1 is composed of 25 nt; G1A2C3G4A5C6G7G8 at the 5′-end and C18C19G20T21T22G23T24C25 at the 3′-end are complementary to each other.

The DNA1–Cd^2+^ complex structure was refined at atomic resolution (2.1 Å) with final *R*_work_ and *R*_free_ values of 23.2% and 27.4%, respectively (Table 1). The structure belongs to the *C*2 space group, containing three DNA1–Cd^2+^ complexes (complexes A, B and C) per asymmetric unit. Figure 1B depicts that each DNA1 molecule can be divided into two regions, namely the stem region and the CBL-loop region, which is formed by G9T10T11C12A13C14A15G16T17 in the middle of DNA1. The stem is formed by C3G4A5C6G7G8 and C18C19G20T21T22G23. T24 is well defined in complex A (Figure 1B) and complex B. However, instead of pairing with A2, the nucleobase of T24 gets inserted into the minor grove and interacts with symmetry-related DNA molecules. T24 of complex C and C25 of all three complexes are disordered.

### Cd^2+^ is coordinated with G9, C12 and G16 of the CBL-loop

**Figure 2.**
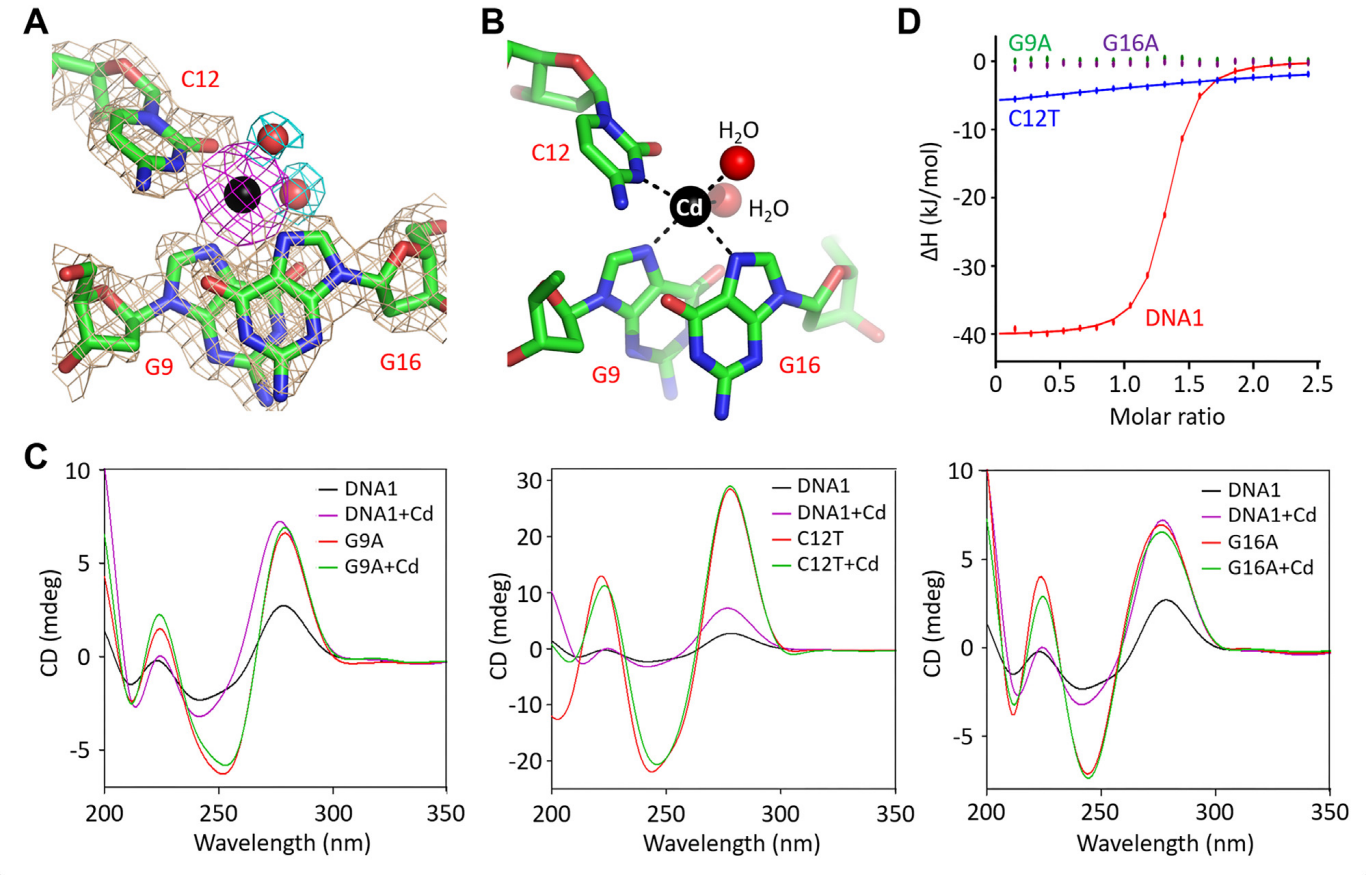
Coordination of Cd^2+^ by the CBL-loop. (**A**) The 2*F*_o_–*F*_c_ electron density maps (contour level, 1.5*σ*) of Cd^2+^ and the coordinating nucleotides. (**B**) The detailed coordination of the Cd^2+^. (**C**) Comparison of the CD spectra of the native DNA1 and the G9A, C12T and G16A variants. (**D**) Comparison of Cd^2+^ binding by the native DNA1 and the G9A, C12T and G16A variants by ITC assays. In panels (A) and (B), Cd^2+^ ion and the Cd^2+^-coordinating water molecules are shown as spheres in black and red, respectively.

Analysis of structural superposition indicates that the orientation between the stem and the CBL-loop is changeable; however, the overall folding of the CBL-loop is highly conserved (Supplementary Figure S1). The root-mean-square deviation (RMSD) value between the CBL-loops of complexes A and C is 0.50 Å, and is only 0.35 Å between complexes A and B. The CBL-loop adopts one double-twisted conformation with a sharp turn at both T10 and T17 sites (Figure 1B). As supported by the clear 2*F*_o_–*F*_c_ electron density maps, each CBL-loop binds one Cd^2+^ ion (Figure 2A). The Cd^2+^ ion mainly coordinates with the N7 atom of G9, the N3 atom of C12 and the N7 atom of G16. Moreover, it also coordinates with two water molecules, which further stabilizes the conformation of the Cd^2+^ ion (Figure 2B). The coordinating mode of Cd^2+^ is very different from that of Hg^2+^, Pb^2+^ and Ag^+^ observed in their aptamer structures (44–50). Compared to Hg^2+^ and Ag^+^, the average coordinating distance (2.4 Å) of Cd^2+^ is slightly longer; however, it is ∼0.2 Å shorter than the Pd^2+^-coordinating distance.

The G9, C12 and G16 nucleobases adopt *anti*-conformations (Figure 2B). Among them, G9 and G16 nucleobases are parallel to each other, forming hydrophobic stacking interactions. C12 points toward G9 and G16 from the minor groove side; its nucleobase is roughly perpendicular to that of G9 and G16. In order to test the functional importance of G9, C12 and G16, systematic mutation was carried out on DNA1 (Supplementary Table S1) and CD spectra were analyzed (Figure 2C and Supplementary Figure S2). In the absence of Cd^2+^, the CD spectra of the native DNA1 show one negative peak at 240 nm and one positive peak at 275 nm. Addition of Cd^2+^ leads to a slightly decreasing peak at 240 nm and a strongly increasing peak at 275 nm. Similarly to the native DNA1, all DNA1 variants with either G9, C12 or G16 mutation show a positive peak at 275 nm. However, in contrast to the native DNA1, the addition of Cd^2+^ does not impact the CD spectrum of any DNA1 variant.

Besides CD analysis, ITC analysis was also performed for the native DNA1 and the G9A, C12T and G16A variants (Table 2, Figure 2D and Supplementary Figure S3). The native DNA1 could bind very tightly with Cd^2+^ ions, and the value of the equilibrium dissociation constant (*K*_D_) is 0.340 ± 0.017 μM. No detectable Cd^2+^-binding affinity could be observed for either G9A or G16A variant. The C12T variant possesses very weak Cd^2+^-binding ability; its *K*_D_ value (66.1 ± 113 μM) is 194-fold higher than that of the native DNA1. Taken together, these observations confirm that G9, C12 and G16 all play a critical role in Cd^2+^ recognition by DNA1 aptamer.

### Effect of T11–A15 pair on Cd^2+^ binding by the aptamer

**Figure 3.**
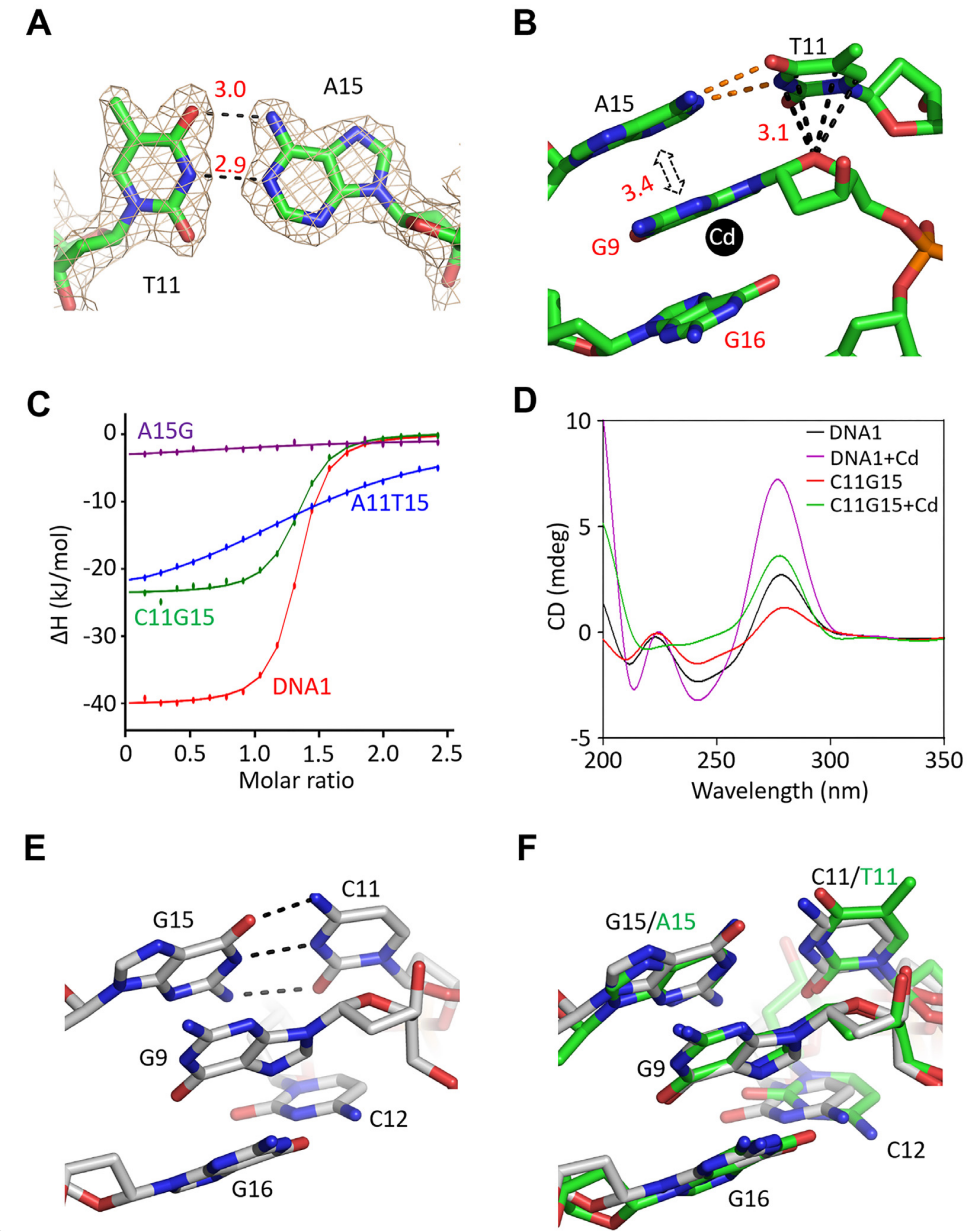
Functional analysis of the CBL-loop T11 and A15 pairing. (**A**) The 2*F*_o_–*F*_c_ electron density maps (contour level, 1.5*σ*) of the T11–A15 pair in the native DNA1–Cd^2+^ structure. (**B**) The detailed interactions between the nucleotide G9 and the T11–A15 pair. (**C**) Comparison of Cd^2+^ binding by the native DNA1 and the A15G, A11T15 and C11G15 variants by ITC assays. (**D**) Comparison of the CD spectra of the native DNA1 and the C11G15 variant. (**E**) Conformations of G9, C11 and G15 in the C11G15–Cd^2+^ complex structure. (**F**) Superposition of the local structures in the native DNA1–Cd^2+^ and the C11G15–Cd^2+^ complex structures. C atoms are colored in green and white in the native aptamer and the variant structures, respectively.

Unlike G9, C12 and G16, other nucleotides of the CBL-loop do not participate in direct Cd^2+^ coordination. As supported by the clear 2*F*_o_–*F*_c_ electron density maps, T11 and A15 are well ordered in the structure (Figure 3A). T11 and A15 form Watson–Crick (W–C) pairing. The bond length of hydrogen bond (H-bond) between the N3 atom of T11 and the N1 atom of A15 is 2.9 Å, and it is 3.0 Å between the O4 of T11 and the N6 of A15. The six-member ring of the T11 nucleobase forms O–π stacking interaction with the O4′ atom of G9 sugar pucker. The average stacking distance is only 3.1 Å, indicating that the interaction is very strong. The G9 and A15 nucleobases are parallel to each other, forming extensive hydrophobic stacking interactions with an average distance of 3.4 Å (Figure 3B).

To investigate whether the T11–A15 pairing is functionally important, in this study, one A15G variant, in which the nucleotide A15 was substituted with G15, was first synthesized. Supplementary Figure S4A depicts that the CD spectrum of the A15G variant is virtually identical in the absence and presence of Cd^2+^. Next, three DNA1 variants with the T11–A15 base pair replaced with other W–C pairs were designed (Supplementary Table S1). Similarly to the A15G variant, the addition of Cd^2+^ does not show obvious impact on the CD spectrum of the A11T15 variant (Supplementary Figure S4A). ITC analysis confirms that the Cd^2+^-binding affinities of the A15G and A11T15 variants (Table 2, Figure 3C and Supplementary Figure S4B and C) are significantly weaker than that of the native DNA1.

Compared to the G11C15 variant (Supplementary Figure S4A), the addition of Cd^2+^ causes a more noticeable increase in the 275 nm peak of the C11G15 variant (Figure 3D). Furthermore, the ITC analysis reveals that the *K*_D_ value (0.515 ± 0.074 μM) of the C11G15 variant is only 1.5-fold weaker than that of the native DNA1 (Figure 3C and Supplementary Figure S4D). In order to further confirm that the C11G15 variant could bind Cd^2+^, its structure was solved in the presence of Cd^2+^. The structure belongs to the *P*2_1_ space group and contains four C11G15–Cd^2+^ complexes per asymmetric unit. The overall folding of the C11G15 variant is similar to that of the native DNA1 structure, in particular, in the CBL-loop region (Supplementary Figure S5). C11 and G15 form regular W–C base pairs (Figure 3E). Structural superposition (Figure 3F) shows that the C11–G15 pair can mimic the T11–A15 pair in stabilizing the G9 nucleotide, which adopts almost identical conformation in the C11G15–Cd^2+^ and the native DNA1–Cd^2+^ complex structures.

### T10 and T17 are important for the folding of the aptamer

**Figure 4.**
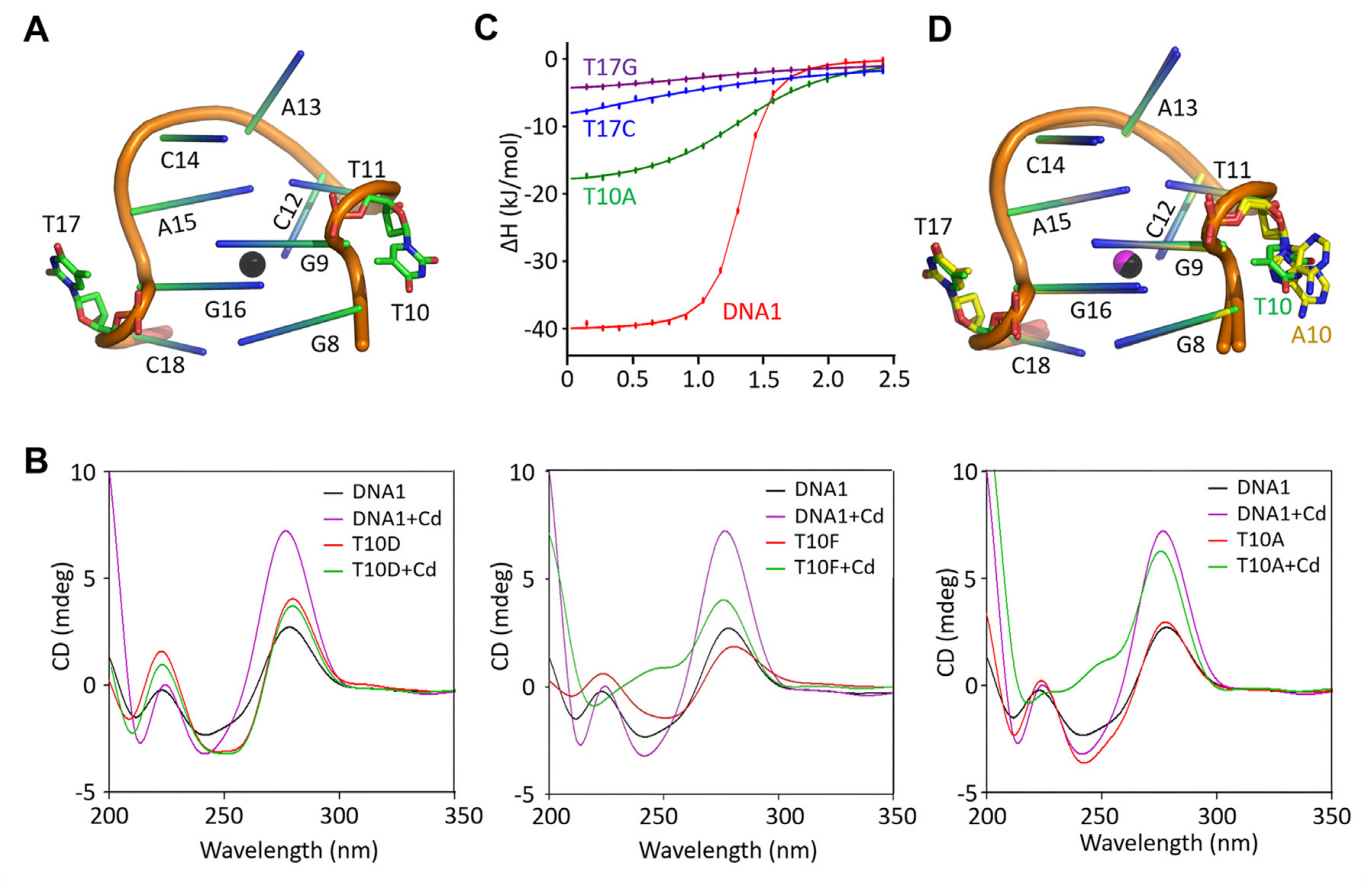
Characterization of the T10 and T17 nucleotides of the CBL-loop. (**A**) The detailed conformations of T10 and T17 in the native DNA1–Cd^2+^ structure. (**B**) Comparison of the CD spectra of the native DNA1 and the T10D, T10F and T10A variants. (**C**) Comparison of Cd^2+^ binding by the native DNA1 and the T10A, T17C and T17G variants by ITC assays. (**D**) Superposition of the CBL-loops in the native DNA1–Cd^2+^ and the T10A–Cd^2+^ complex structures. T10 and T17 are highlighted as sticks in atomic colors (C, green; N, blue; O, red; P, orange) in the native DNA1–Cd^2+^ structure. In the T10A–Cd^2+^ structure, C atoms of A10 and T17 are colored in yellow.

Both T10 and T17 nucleotides were found to be severely twisted in the DNA1–Cd^2+^ structure (Figure 4A) and the C11G15–Cd^2+^ structure (Supplementary Figure S5). Instead of the nucleotides within the same CBL-loop, the nucleobases of T10 and T17 mainly formed hydrophobic stacking or H-bond interaction with symmetry-related aptamer molecules. To investigate the function of T10, one deletion variant, namely T10D, was constructed herein and the nucleotide T10 was deleted. Figure 4B illustrates that the addition of Cd^2+^ does not impact the CD spectrum of T10D, indicating that T10 is indispensable for Cd^2+^ binding by DNA1.

For the further clarification of the function of T10, one T10F variant, in which T10 was replaced with an abasic site, was designed herein. Similarly to the native DNA1, the addition of Cd^2+^ leads to a clear increase in the 275 nm peak of the CD spectrum of T10F (Figure 4B), indicating that the nucleobase of T10 is not critical for Cd^2+^ binding by DNA1. We then wondered whether T10 could be replaced with other regular nucleotides. To this end, in this study, T10A, T10C and T10G variants of DNA1 were synthesized. Compared to the T10C and T10G variants (Supplementary Figure S6A), the CD spectrum of T10A was more sensitive to Cd^2+^ (Figure 4B). Though not as strong as the native DNA1, ITC analysis confirmed that the T10A variant could bind Cd^2+^ (Figure 4C and Supplementary Figure S6B); the *K*_D_ value of T10A was found to be 3.83 ± 0.282 μM.

The binding of Cd^2+^ by the T10A variant could be further supported by the T10A–Cd^2+^ complex structure. The structure belongs to the *C*2 space group (Table 1) and contains two T10A–Cd^2+^ complexes per asymmetric unit. The low RMSD value (0.55 Å) indicates that the overall folding of the two T10A–Cd^2+^ complexes is very similar (Supplementary Figure S7A). The relative orientations between the stem and the CBL-loop regions are different in the T10A–Cd^2+^ complex and the native DNA1–Cd^2+^ structure (Supplementary Figure S7B). However, the conformations of the CBL-loops are very similar (Figure 4D); the RMSD value between the CBL-loops of the two structures is ∼0.33 Å. The nucleobase of A10 shows two alternative conformations. Similarly to T10 in the native DNA1–Cd^2+^ structure, A10 points away from any nucleotide within the CBL-loop.

In order to verify the function of T17, several DNA1 variants with T17 deleted or mutated were designed (Supplementary Table S1) and CD analysis was carried out (Supplementary Figure S8A). The CD spectra of the T17D and T17F variants are similar to those of the T10D and T10F variants, respectively. Compared to the T17A variant, the spectra of the T17G and T17C variants are more sensitive to Cd^2+^. However, ITC analysis exhibits that the Cd^2+^-binding affinities of the T17G and T17C variants are significantly weaker than that of the native DNA1 (Table 2 and Supplementary Figure S8B and C). Taken together, these observations indicate that T10 and T17 mainly play a structural role in the folding of DNA1, but replacing T10 and T17 with other nucleotides may alter the structure and affect the Cd^2+^ binding by DNA1.

**Table 2.** *K*_D_ values for Cd^2+^ binding by DNA1 and variants

Name	*K*_D_ (μM)	Name	*K*_D_ (μM)
DNA1	0.340 ± 0.017	A13G	3.03 ± 0.315
G8A	82.1 ± 237	C14T	0.317 ± 0.007
C8G18	2.33 ± 0.301	A15G	25.8 ± 80.4
A8T18	0.143 ± 0.005	G16A	ND
G9A	ND	T17G	13.3 ± 9.11
T10A	3.83 ± 0.282	T17C	46.5 ± 58.0
A11T15	12.5 ± 1.42	T22C	0.373 ± 0.011
C11G15	0.515 ± 0.074	DNA2	0.274 ± 0.028
C12T	66.1 ± 113		

ND, not detectable.

### A13 and C14 are changeable for Cd^2+^ binding by the aptamer

**Figure 5.**
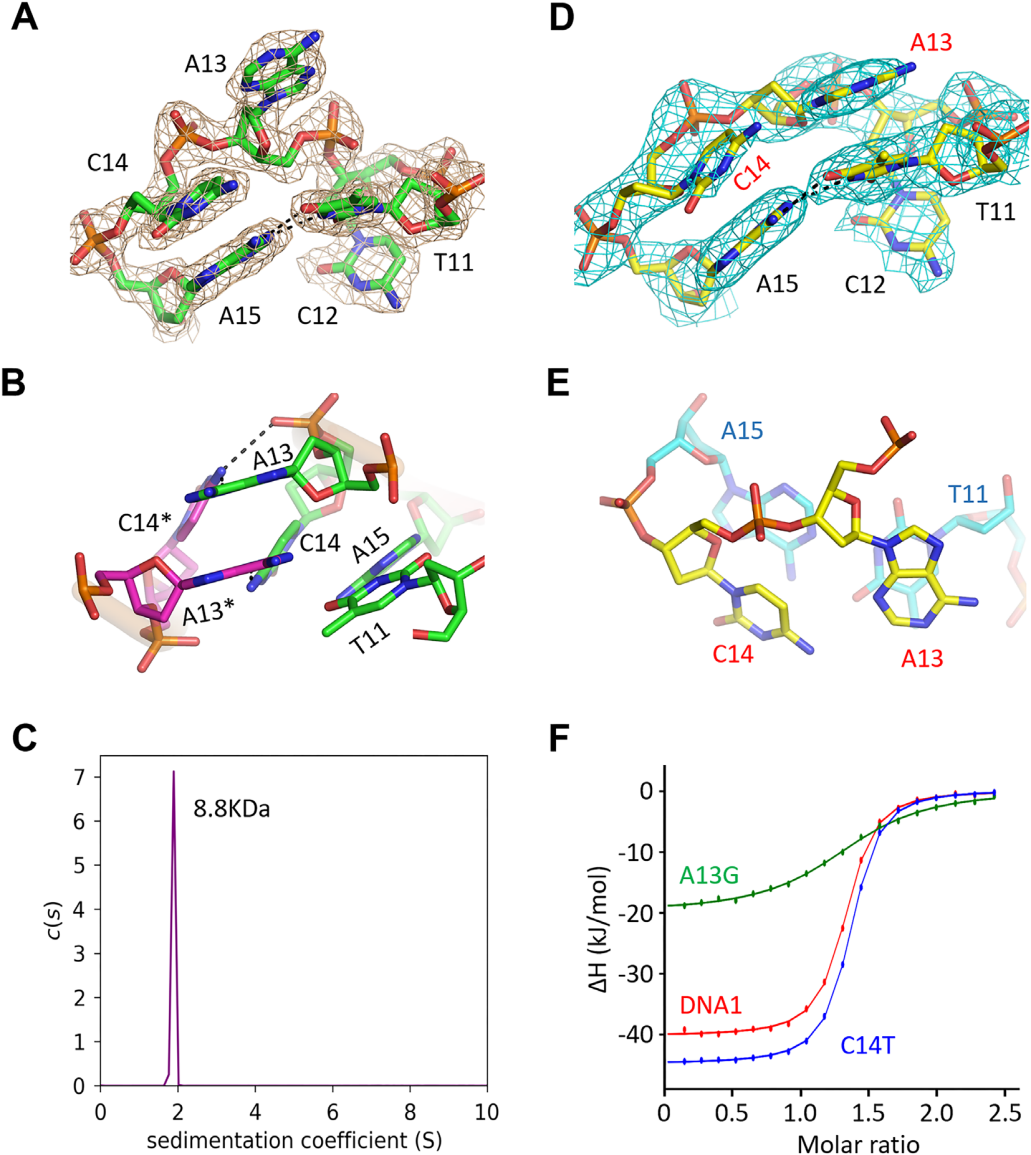
Conformational change and impacts of A13 and C14 on Cd^2+^ binding. (**A**) The 2*F*_o_–*F*_c_ electron density maps (contour level, 1.5*σ*) of A13 and C14 in the native DNA1–Cd^2+^ structure. (**B**) The detailed interactions between A13 and C14 of two DNA1 molecules in the crystal lattice of the native DNA1–Cd^2+^ structure. (**C**) AUC analysis for the native DNA1–Cd^2+^ complex. (**D**) The 2*F*_o_–*F*_c_ electron density maps (contour level, 1.5*σ*) of A13 and C14 in the T22C–Cd^2+^ complex structure. (**E**) The detailed conformations of A13, C14 and neighboring nucleotides in the T22C–Cd^2+^ complex structure. (**F**) Comparison of Cd^2+^ binding by the native DNA1 and the T10A, T17C and T17G variants by ITC assays.

The packing of the T10A–Cd^2+^ and the native DNA1–Cd^2+^ complexes is very different in their crystal lattices. Compared to the native DNA1–Cd^2+^ structure, the distance between the A13 nucleotides of the pseudo-dimer is ∼7.3 Å longer in the T10A–Cd^2+^ structure (Supplementary Figure S9). However, structural superposition shows that the overall folding of the CBL-loop and the conformation of A13 are virtually identical in the two structures (Figure 4D and Supplementary Figure S7B). The clear 2*F*_o_–*F*_c_ electron density maps clearly indicate that A13 and the neighboring nucleotide C14 are well ordered in the native DNA1–Cd^2+^ complex structure (Figure 5A). A13 and C14 of two symmetry-related DNA1 molecules interact with each other (Figure 5B and Supplementary Figure S10A). The A13 and A13* nucleobases form extensive hydrophobic stacking interactions. The stacking interactions are also formed between the C14 and C14* nucleobases. The conformations of A13 and C14 are further stabilized by their H-bond interactions with C14* and A13*, respectively.

To investigate whether A13 and C14 affect Cd^2+^ binding by DNA1, the CD spectra of the DNA1 variants with either A13 or C14 mutation were measured in this study (Supplementary Table S1). Supplementary Figure S11A exhibits that the addition of Cd^2+^ can cause an increase in the 275 nm peak for all the A13G, A13C, A13T, C14A, C14G and C14T variants, indicating that the interactions between the symmetry-related A13 and C14 are not critical for Cd^2+^ binding. To further clarify the oligomerization state of DNA1 in solution, more crystallization trials were performed herein. Though we failed to obtain any crystal for DNA1 with either A13 or C14 mutated, one T22C variant structure in the presence of Cd^2+^ was successfully solved (Table 1). The structure belongs to the *P*4_3_2_1_2 space group and contains one T22C–Cd^2+^ complex per asymmetric unit. Packing of the T22C–Cd^2+^ complex in the crystal lattice is different from that in all other structures. Supplementary Figure S10B illustrates that each T22C–Cd^2+^ complex is surrounded by several T22C molecules; however, none of them interact with A13 or C14. Compared to the native DNA1–Cd^2+^ structure, the nucleobase of A13 undergoes 40° rotation in the T22C–Cd^2+^ complex structure (Supplementary Figure S10C).

Consistent with the T22C–Cd^2+^ complex structure, the AUC analysis also confirms that DNA1–Cd^2+^ complex exists as a monomer in solution (Figure 4C). The sedimentation coefficient value of the complex is 1.891 S, and the calculated molecular weight (8814 Da) matches well with the theoretical one (7786 Da). Unlike all other structures, A13 does not interact with symmetry-related C14 in the T22C–Cd^2+^ complex structure; the conformation of the CBL-loop in the T22C–Cd^2+^ complex should be the closest to the one in solution. Figure 5D exhibits that A13 is well ordered in the T22C–Cd^2+^ structure, supported by the clear 2*F*_o_–*F*_c_electron density maps. The nucleobase of A13 stacks with T11 from the same CBL-loop, forming extensive hydrophobic interactions. C14 in the T22C–Cd^2+^ structure does not interact stably with any nucleotide (Figure 5D and E). Both the weak electron density maps and high *B*-factor indicate that the conformation of C14 is dynamic. Consistent with structural observations, ITC analysis confirms that the Cd^2+^-binding affinity of the A13G variant is 8.9-fold weaker than that of the native DNA1. However, the *K*_D_ values of the T14C variant and the native DNA1 are comparable (Table 2, Figure 5F and Supplementary Figure S11B and C).

### G8–C18 pair of the stem is important for Cd^2+^ binding

**Figure 6.**
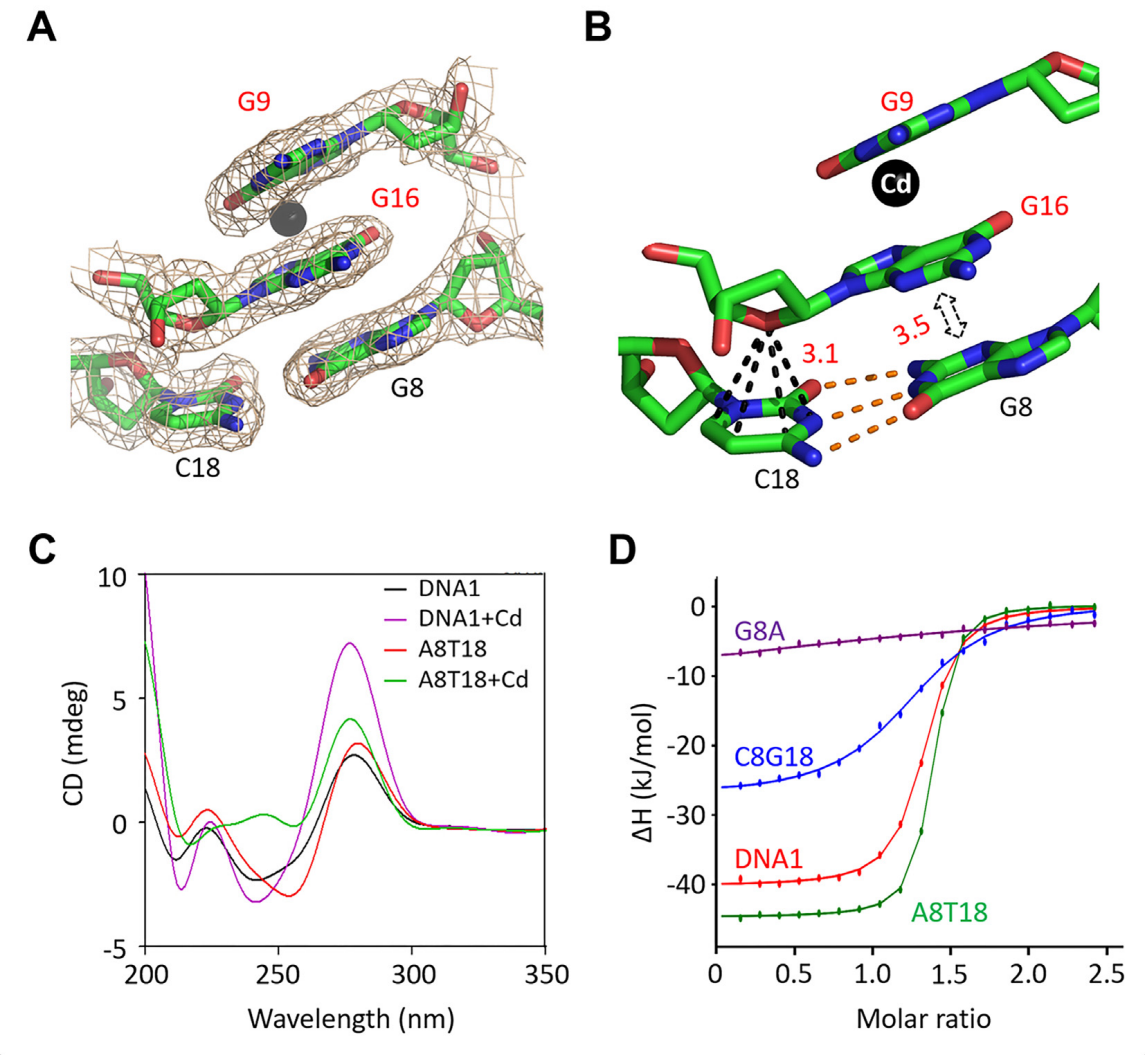
Functional analysis of the G8–C18 pair of the stem. (**A**) The 2*F*_o_–*F*_c_ electron density maps (contour level, 1.5*σ*) of G16 and G8–C18 pair in the native DNA1–Cd^2+^ structure. (**B**) The detailed conformation and interactions between the nucleotide G16 and the G8–C18 pair. (**C**) Comparison of the CD spectra of the native DNA1 and the A8T18 variant. (**D**) Comparison of Cd^2+^ binding by the native DNA1 and the A8T18, C8G18 and G8A variants by ITC assays.

G8 and C18 of DNA1 form one regular W–C base pair, which is also the last pair of the stem. Neither G8 nor C18 is involved in Cd^2+^ coordination; however, they form extensive interactions with the Cd^2+^-coordinating nucleotide G16 (Figure 6A and B). The nucleobases of G8 and G16 are parallel to each other, forming extensive hydrophobic stacking interactions, and the average stacking distance is 3.5 Å. The six-member ring of the C18 nucleobase forms an O–π stacking interaction with the O4′ atom of G16 sugar pucker; the average stacking distance is only 3.1 Å. To investigate whether the G8–C18 pair plays a role in Cd^2+^ binding by DNA1, four variants, including G8A, C8G18, A8T18 and T8A18, were synthesized in this study (Supplementary Table S1). The addition of Cd^2+^ does not show obvious impacts on the CD spectrum of the G8A variant; however, it can lead to some shifting and increasing of the 275 nm peaks of the C8G18, T8A18 and A8T18 variants (Figure 6C and Supplementary Figure S12A). ITC analysis further reveals that the Cd^2+^-binding affinities of the G8A and C8G18 variants are weaker than that of the native DNA1 (Supplementary Figure S12B and C). However, compared with the native DNA1, the Cd^2+^-binding affinities of the A8T18 variant are 2.4-fold stronger (Table 2, Figure 6D and Supplementary Figure S12D). The relative orientation between G16 and the G8–C18 pair is very similar to that between G9 and the T11–A15 pair (Figure 3B). Similarly to the T11–A15 pair, the G8–C18 pair is likely involved in the proper folding and stabilization of the CBL-loop.

### Characterization of the G4–T22 pair and the length of the stem

**Figure 7.**
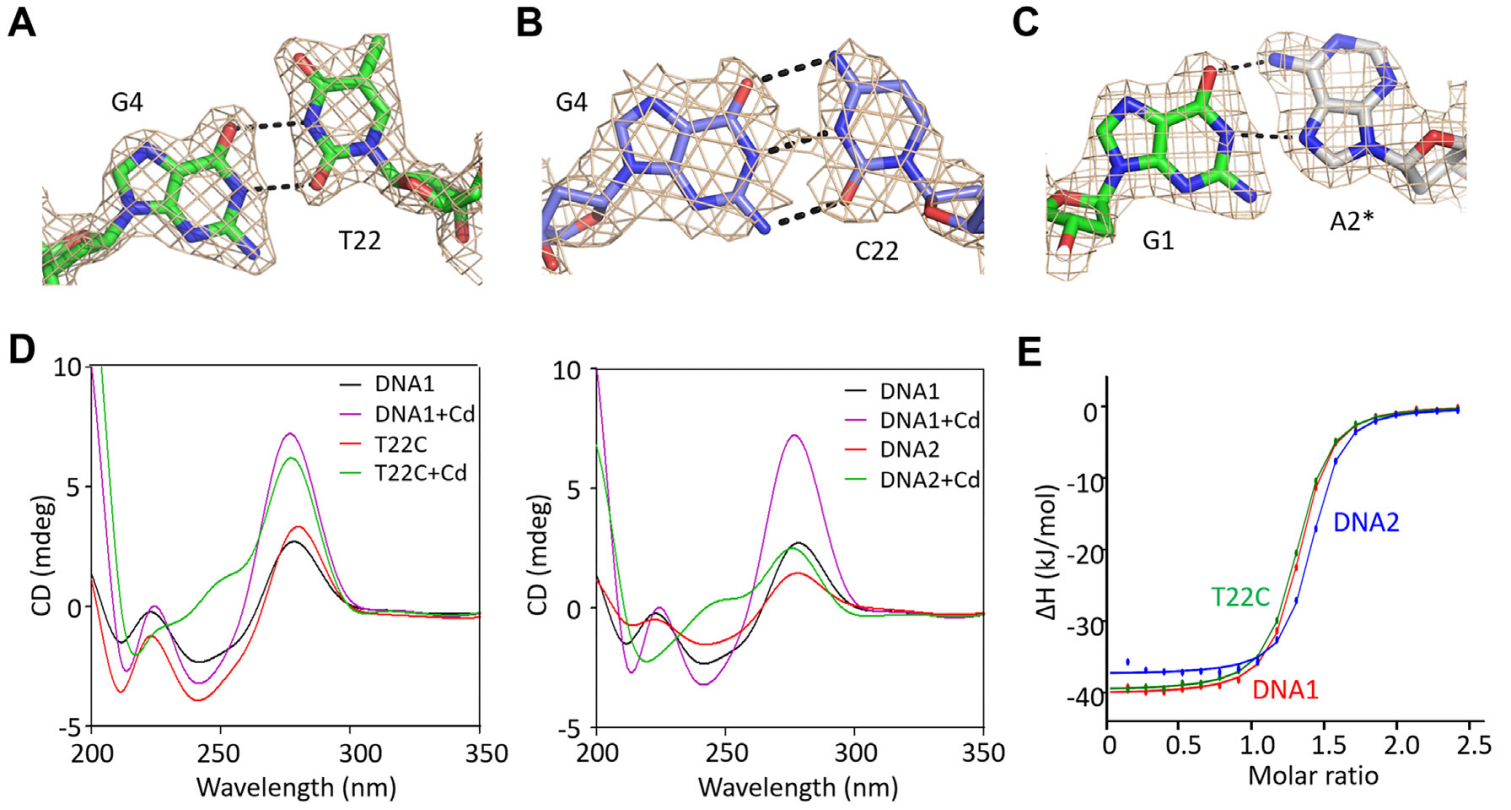
Functional characterization of the G4–T22 pair and length of the aptamer. (**A**) The 2*F*_o_–*F*_c_ electron density maps of G4 and T22 in the native DNA1–Cd^2+^ structure. (**B**) The 2*F*_o_–*F*_c_ electron density maps of G4 and C22 in the T22C–Cd^2+^ structure. (**C**) The 2*F*_o_–*F*_c_ electron density maps and interactions between G1 and A2 of two DNA1 molecules in the native DNA1–Cd^2+^ structure. (**D**) Comparison of the CD spectra of the native DNA1, the T22C variant and DNA2. (**E**) Comparison of Cd^2+^ binding by the native DNA1, the T22C variant and DNA2 by ITC assays. All maps are contoured at 1.5*σ* level.

The DNA1 stem region contains one G and one T nucleotide at the 4th and 22nd positions, respectively. The clear electron density maps support that the G4 and T22 nucleotides are well ordered and form one G–T wobble pair in the native DNA1–Cd^2+^ complex structure (Figure 7A). The G4–T22 wobble pair was also conserved in the T10A–Cd^2+^ and the C11G15–Cd^2+^ complex structures. Different from the native DNA1 and other variants, the nucleotide T22 was replaced by C22 in the T22C variant. In the T22C–Cd^2+^ complex structure, G4 and C22 formed one regular W–C pair (Figure 7B). As mentioned earlier, A13 adopts different conformations in the native DNA1–Cd^2+^ and T22C–Cd^2+^ complex structures; nonetheless, the conformations of other nucleotides of the CBL-loop are virtually identical in the two structures (Supplementary Figure S10C).

The 16 nucleotides in the stem region form eight base pairs in the C11G15–Cd^2+^ (Supplementary Figure S5A), the T10A–Cd^2+^ (Supplementary Figure S7A) and the T22C–Cd^2+^ (Supplementary Figure S10B) complex structures. However, the sugar pucker and the nucleobase of C25 are completely disordered in the native DNA1–Cd^2+^ complex structure (Figure 1B). The nucleobase of C24 is flipped and points away from the duplex. Instead of the regular W–C pair, G1 and A2 of one DNA1 molecule form Hoogsteen interactions with A2* and G1* from another DNA1 molecule in the crystal lattice, respectively. The nucleobases of G1 and G1* adopt normal *anti*-conformation, whereas A2 and A2* exhibit *syn*-conformation (Figure 7C).

In addition to structural analysis, CD and ITC analysis (Figure 7D and E and Supplementary Figure S13) was also performed for the T22C variant and DNA2, which only has six base pairs at the stem region (Supplementary Table S1). Similarly to the native DNA1, the T22C variant shows one negative peak at 240 nm and one positive peak at 275 nm; the addition of Cd^2+^ causes a strong increase in the intensity of the peak at 275 nm. The *K*_D_ values of the T22C variant and the native DNA1 are comparable (Table 2). Compared to DNA1, the CD spectrum of DNA2 is less sensitive to Cd^2+^, but ITC analysis indicates that the Cd^2+^-binding affinity of DNA2 is even slightly higher than that of DNA1. Taken together, these observations confirm that the G4–T22 wobble pair in DNA1 can be replaced with regular W–C pairs and the length of the stem can also be shortened, providing another important mutation site for designing new Cd^2+^-binding aptamers.

## CONCLUSIONS

In this study, we solved four Cd^2+^-bound aptamer structures, which represent the only Cd^2+^-specific aptamer structures available to date. All these structures show that Cd^2+^ is mainly coordinated with the N7 atom of G9, the N3 atom of C12 and the N7 atom of G16 of the CBL-loop. The thorough structural analysis together with CD spectra and ITC analysis also confirms that the T11–A15 pair of the CBL-loop and the G8–C18 pair of the stem play important roles in Cd^2+^ binding by DNA1, via stabilizing the Cd^2+^-coordinating nucleotides. Although the replacement of the G8–C18 or T11–A15 pair with some W–C pairs impaired the Cd^2+^ binding by the aptamer, the C11G15 variant still exhibited strong Cd^2+^-binding ability and the Cd^2+^-binding affinity of the A8T18 variant was even better than that of the native DNA1. Other nucleotides mainly play a structural role (T10 and T17) or stabilize the proper folding (A13 and C14) of the CBL-loop. Replacement of C14 with T14 does not show strong impact on Cd^2+^ binding by the aptamer; however, replacing T10, A13 and T17 by other nucleotides may alter the structure and affect Cd^2+^ binding. The stem can be formed by all regular W–C pairs and the length of the stem can be further shortened, which eases the synthesis and decreases the cost of the aptamer.

Previously, one Cd^2+^-specific protein structure was reported, which revealed the two different Cd^2+^-binding modes (58). The Cd^2+^ coordinates with the side chains of one glutamic acid and three histidine residues in mode A, but coordinates with the side chains of three cysteine residues and one asparagine in mode B. The average Cd^2+^-coordinating distances are conserved and the DNA1–Cd^2+^ coordinating mode is very similar to mode A in the protein structure. However, the DNA1–Cd^2+^ coordinating mode is very different from those between aptamers and other heavy metal ions. As observed in their complex structures, Hg^2+^ and Ag^+^ mainly form metallo-base pairs with pyrimidine–pyrimidine (T–T, C–T and C–C) or purine–purine (G–G) mispairs (46–50), whereas Pb^2+^ is recognized by G-quadruplexes (44,59) or DNAzymes (45). In addition to the DNA strand, recognition of Pd^2+^ by DNAzyme also requires one substrate RNA strand. No obvious conformational change was observed for the Pb^2+^-specific DNAzyme in the presence or absence of Pb^2+^, but Ag^+^ coordination could drive large conformational changes to the aptamers. The CBL-loop adopts one compact, double-twisted conformation in all the Cd^2+^-bound aptamer structures (Figure 1B), but no crystal grew for any of these aptamers in the absence of Cd^2+^. Similarly to Ag^+^, these observations indicate that binding with Cd^2+^ can cause large conformational changes to the aptamers. In principle, in addition to the Cd^2+^-specific detectors, these aptamers can also be utilized in the development of novel DNA nanodevices, which are controlled by binding or release of Cd^2+^.

## DATA AVAILABILITY

The atomic coordinates and structural factors have been deposited in the Protein Data Bank with the ID codes 8GZJ, 8GJK, 8GZL and 8GZM for the native DNA1–Cd^2+^, the T10A–Cd^2+^, the C11G15–Cd^2+^ and the T22C–Cd^2+^ complexes, respectively.

## Supplementary Material

gkad239_Supplemental_File
