# Supplementary material for: Crystal structures and identification of novel Cd2+-specific DNA aptamer
Source: Nucleic Acids Res. 2023 Apr 4;51(9):4625–36. doi: 10.1093/nar/gkad239 (PMC10201416; doi:10.1093/nar/gkad239)
Supplement: gkad239_Supplemental_File [file gkad239_supplemental_file.pdf]

Supporting Information

For

Crystal structures and identification of novel Cd^{2+}-specific DNA aptamer

Hehua Liu[1,#], Yanqing Gao[1,#], Johnsi Mathivanan[2,#], Zev Armour-Garb[2], Zhiwei Shao[1], Yixi Zhang[1], Xin Zhao[1], Qiyuan Shao[1], Weizhen Zhang[1], Jie Yang[1], Chulei Cao[1], Huili Li[1], Jia Sheng[2,*], Jianhua Gan[1,*]

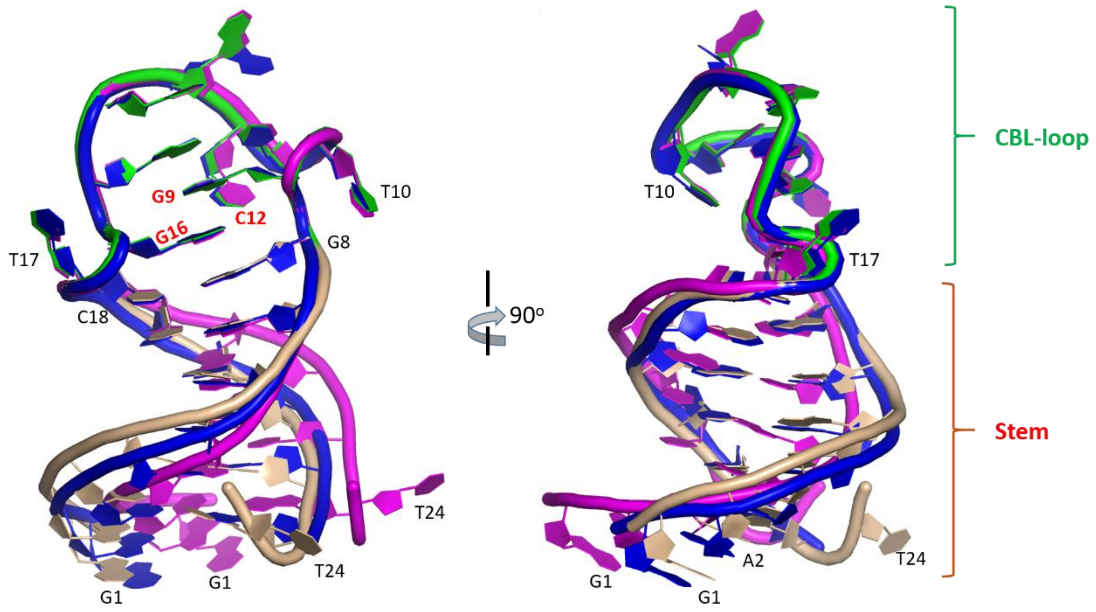

Supplementary Figure S1. Superposition of the three DNA1-Cd^{2+} complexes in the native DNA1 structure. The CBL-loop and the stem in complex A are colored in green and wheat, respectively. Both the CBL-loop and the stem are colored magenta in complex B and blue in complex C.

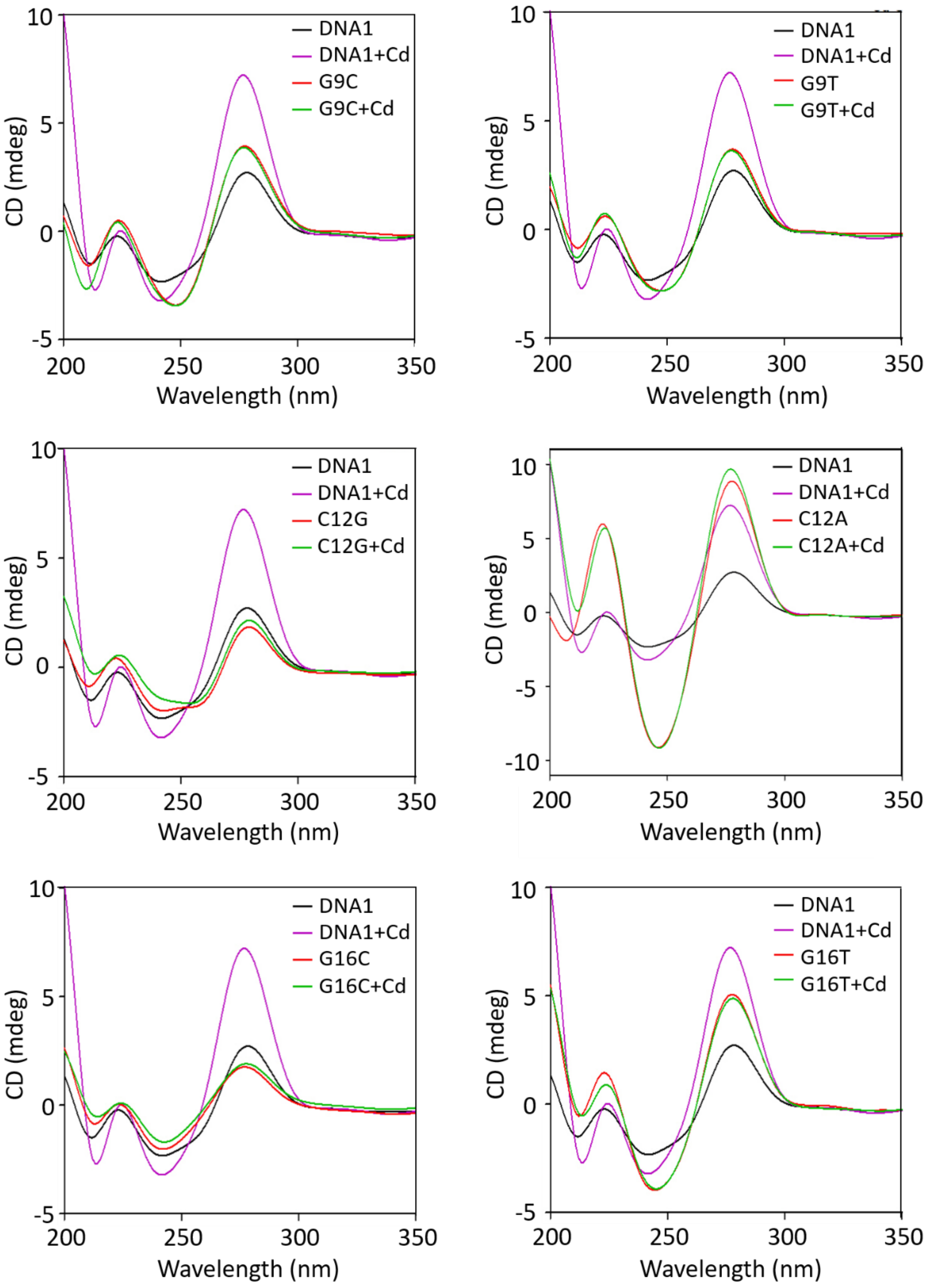

Supplementary Figure S2. Comparison of the CD spectra of the native DNA1 and variants with G9, C12 or G16 mutated.

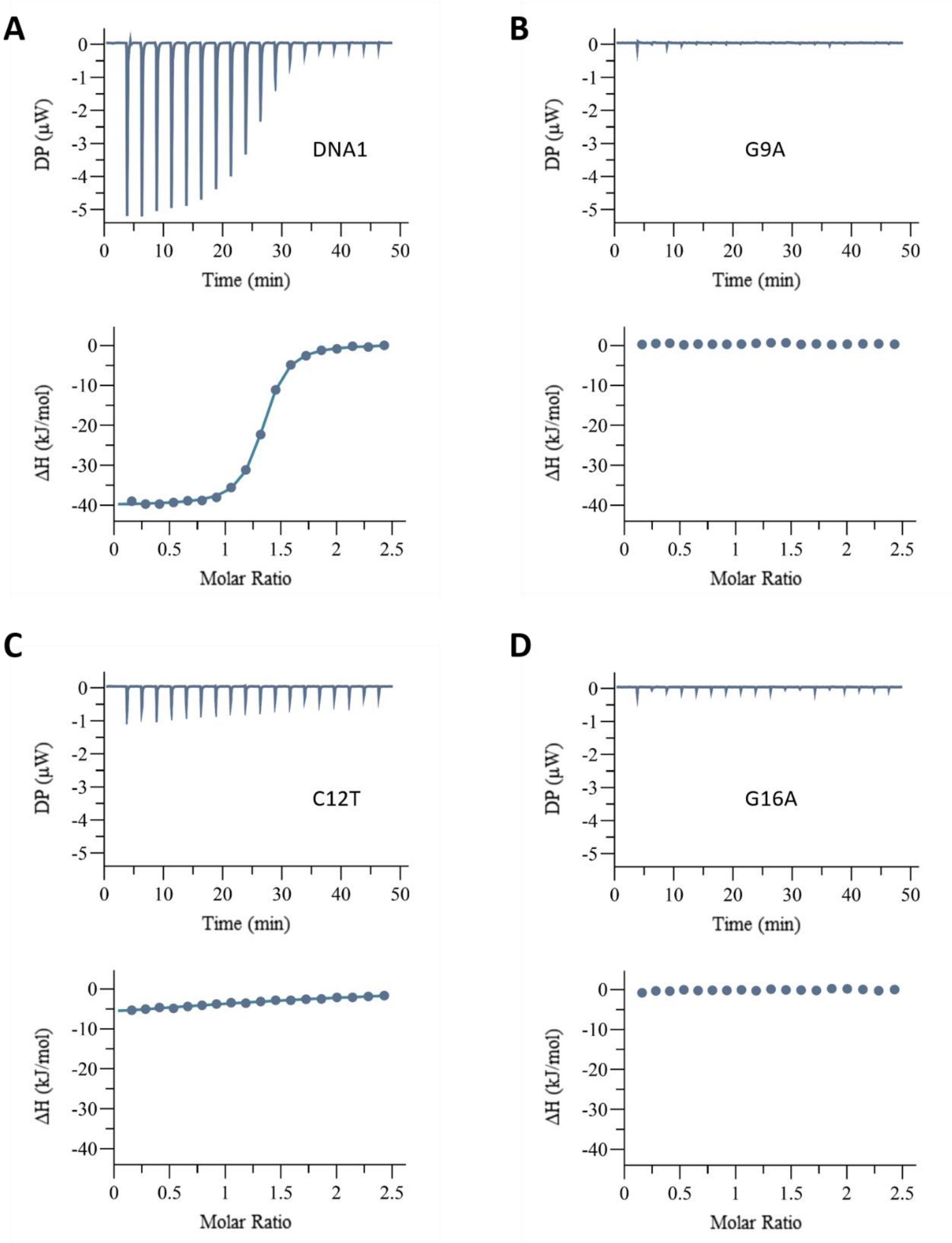

Supplementary Figure S3. ITC analysis showing Cd^{2+} binding by (**A**) the native DNA1, (B) the G9A variant, (C) the C12T variant and (D) the G16A variant, respectively.

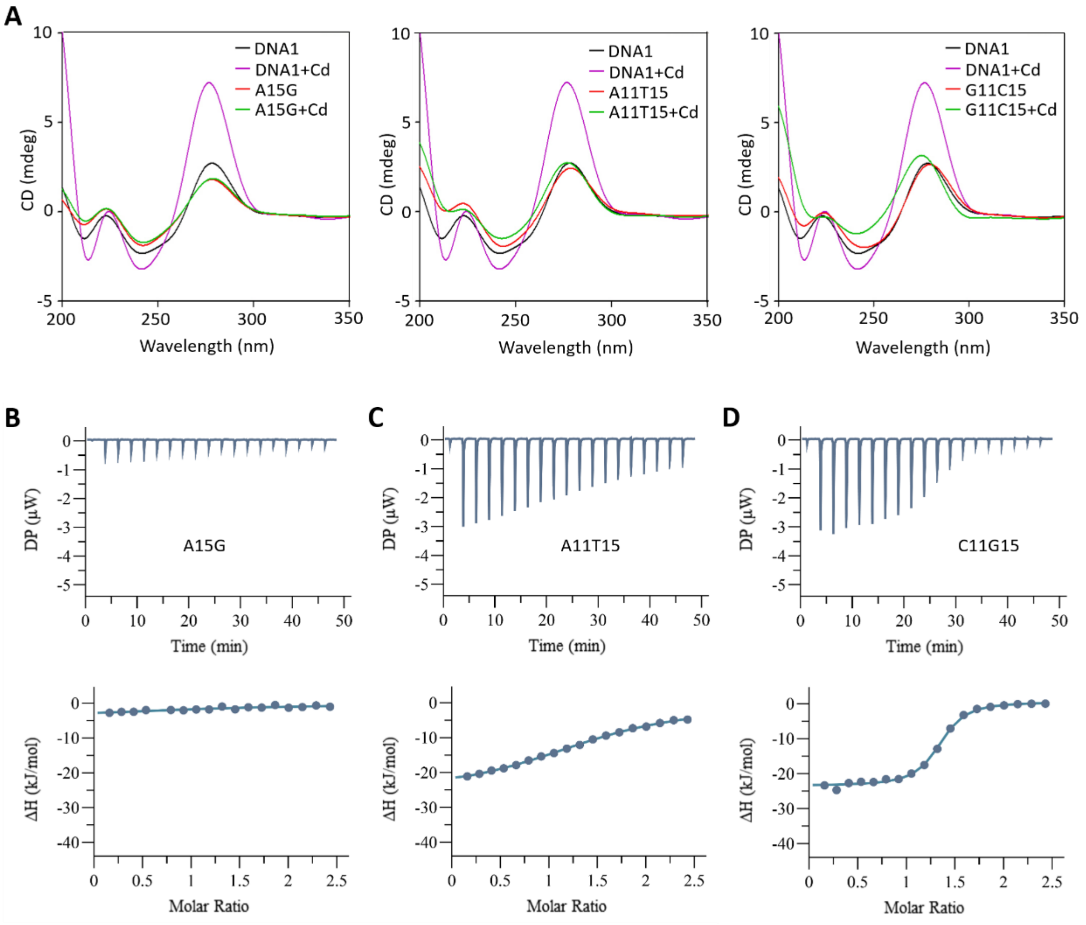

Supplementary Figure S4. (**A**) Comparison of the CD spectra of the native DNA1, the A15G, A11T15 and G11C15 variants. (**B-D**) ITC analysis showing Cd^{2+} binding by the A15G, A11T15 and C11G15 variants, respectively.

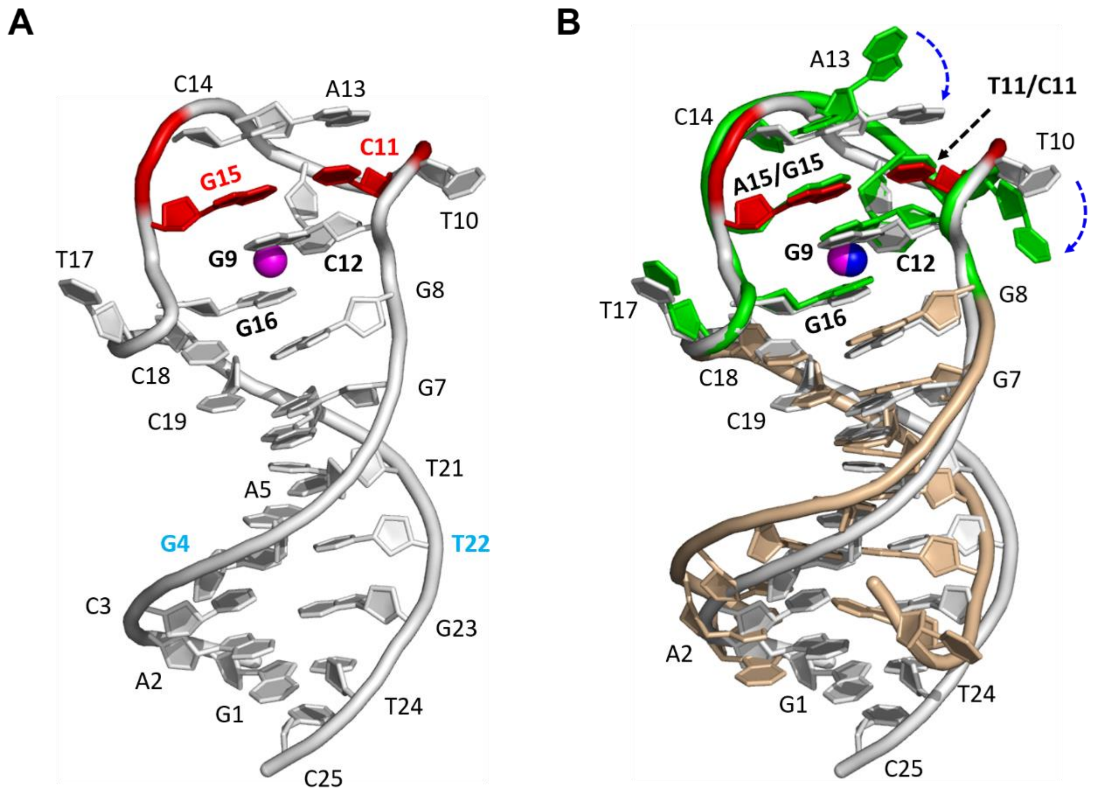

Supplementary Figure S5. (**A**) The overall structure of the C11G15-Cd^{2+} complex. (B) Superposition of the native DNA1-Cd^{2+} and the C11G15-Cd^{2+} complex structures. The CBL-loop, stem and Cd^{2+} are colored green, wheat and blue in the native DNA1-Cd^{2+} structure, respectively.

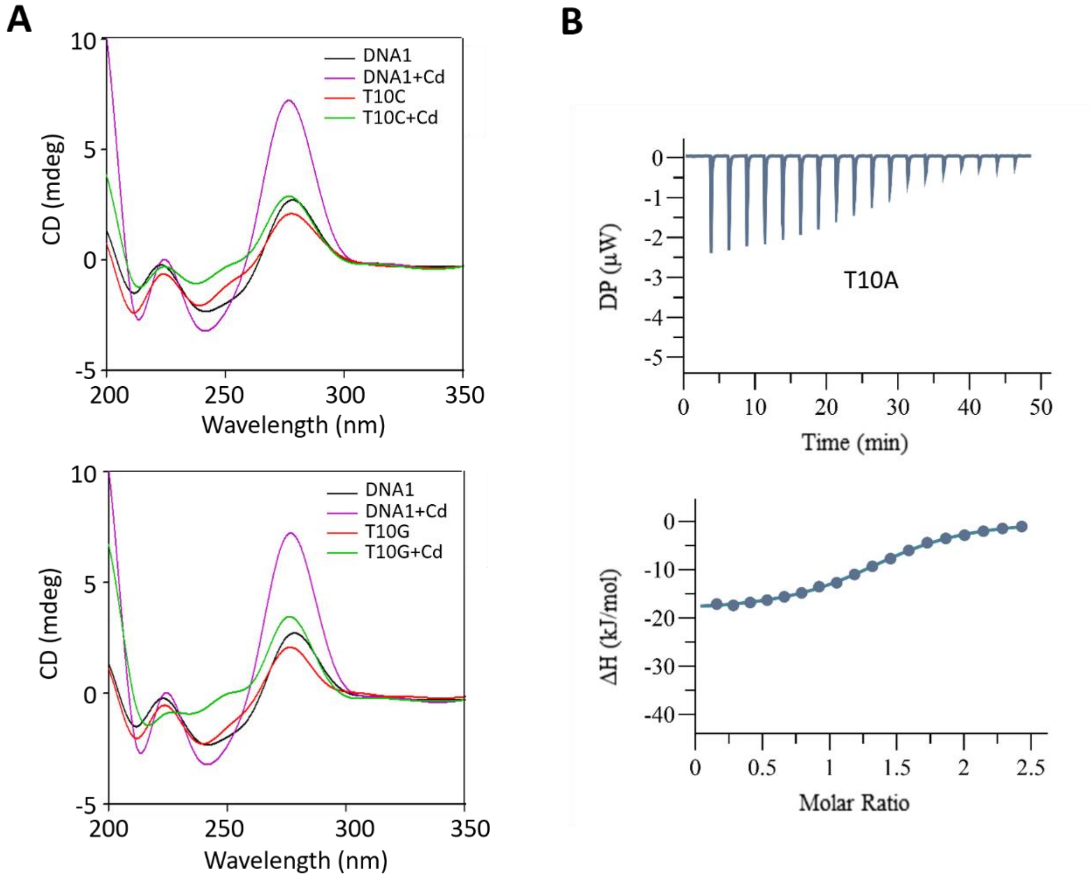

Supplementary Figure S6. (**A**) Comparison of the CD spectra of the native DNA1 and the T10C and T10G variants. (**B**) ITC analysis showing Cd^{2+} binding by the T10A variant.

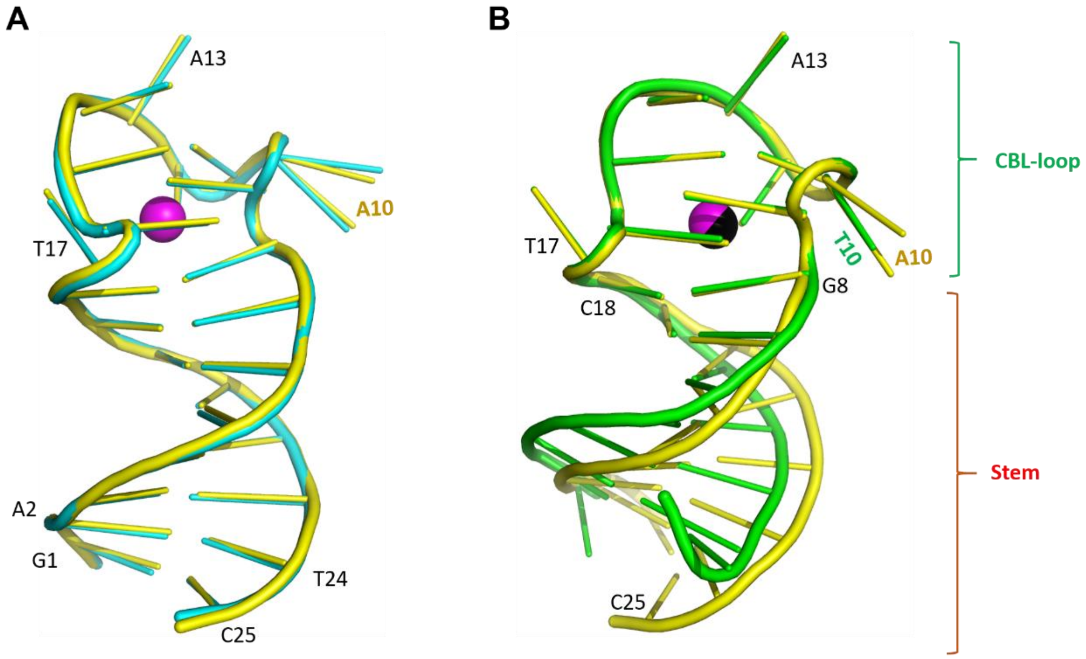

Supplementary Figure S7. (**A**) Superposition of the two T10A-Cd^{2+} complexes in the T10A-Cd^{2+} structure. (**B**) Superposition of the native DNA1-Cd^{2+} (green and black) and the T10A-Cd^{2+} (yellow and magenta) complexes.

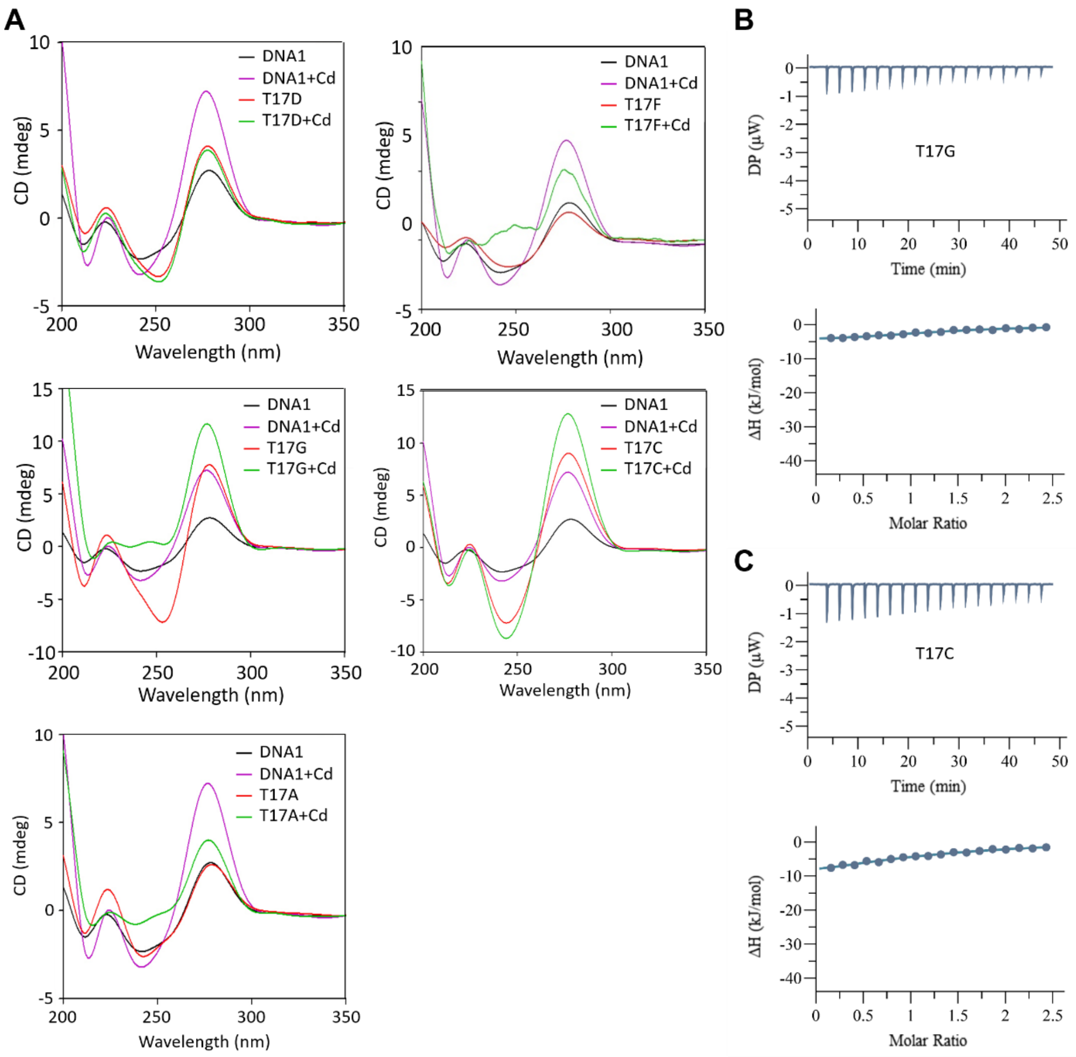

Supplementary Figure S8. (**A**) Comparison of the CD spectra of the native DNA1 and variants with T17 deleted or mutated. (**B-C**) ITC analysis showing Cd^{2+} binding by the T17G and T17C variants, respectively.

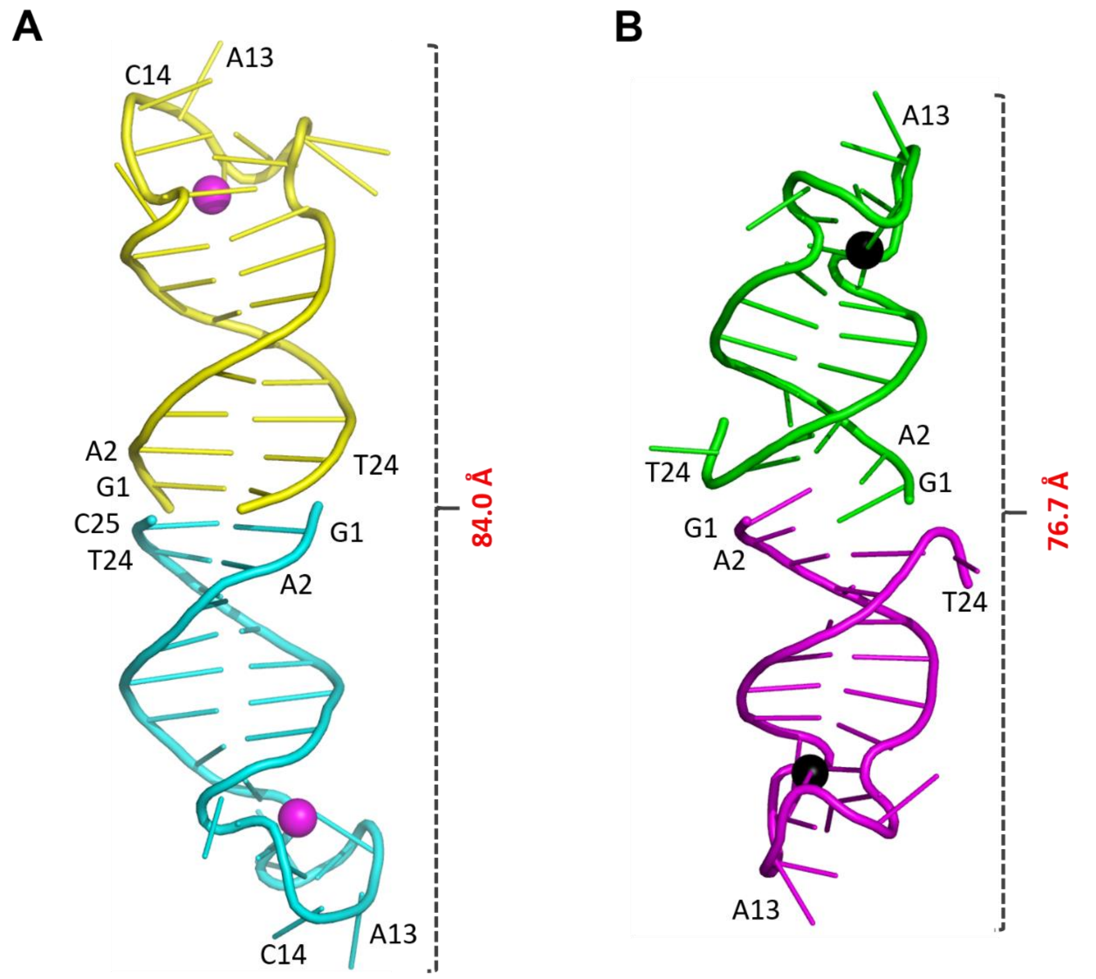

Supplementary Figure S9. (A) Stacking of two T10A molecules in the crystal lattice of the T10A-Cd^{2+} structure. (B) Stacking of two DNA1 molecules in the crystal lattice of the native DNA1-Cd^{2+} structure.

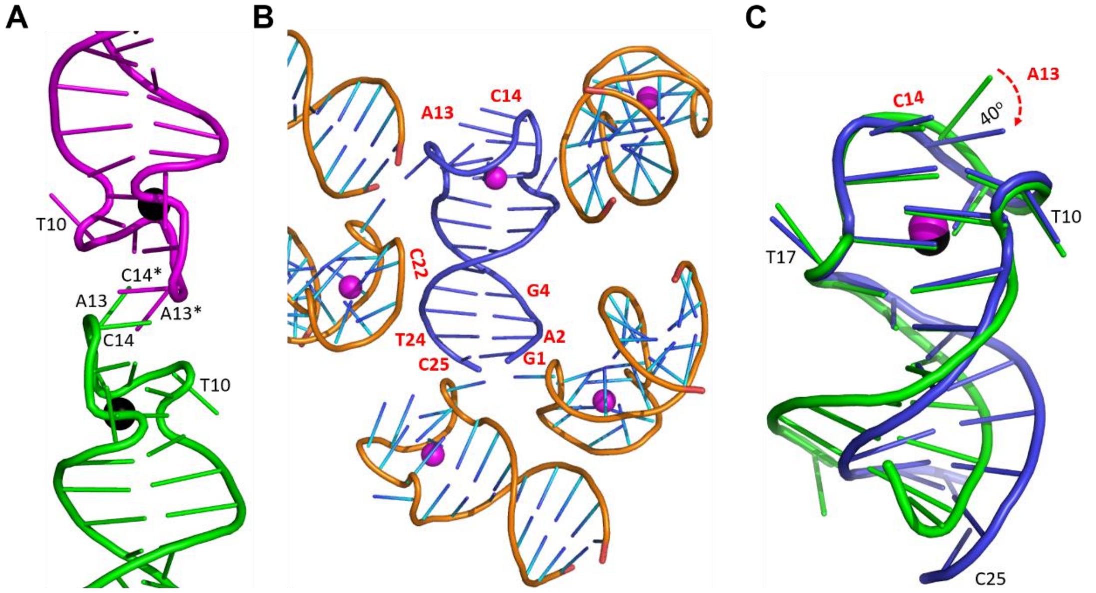

Supplementary Figure S10. (**A**) Stacking of two DNA1 molecules in the crystal lattice of the native DNA1-Cd^{2+} structure. (**B**) Packing of the T22C variant in the crystal lattice of the T22C-Cd^{2+} structure. (**C**) Superposition of the CBL-loops in the native DNA1-Cd^{2+} and the T22C-Cd^{2+} complex structures, which are colored green and blue, respectively.

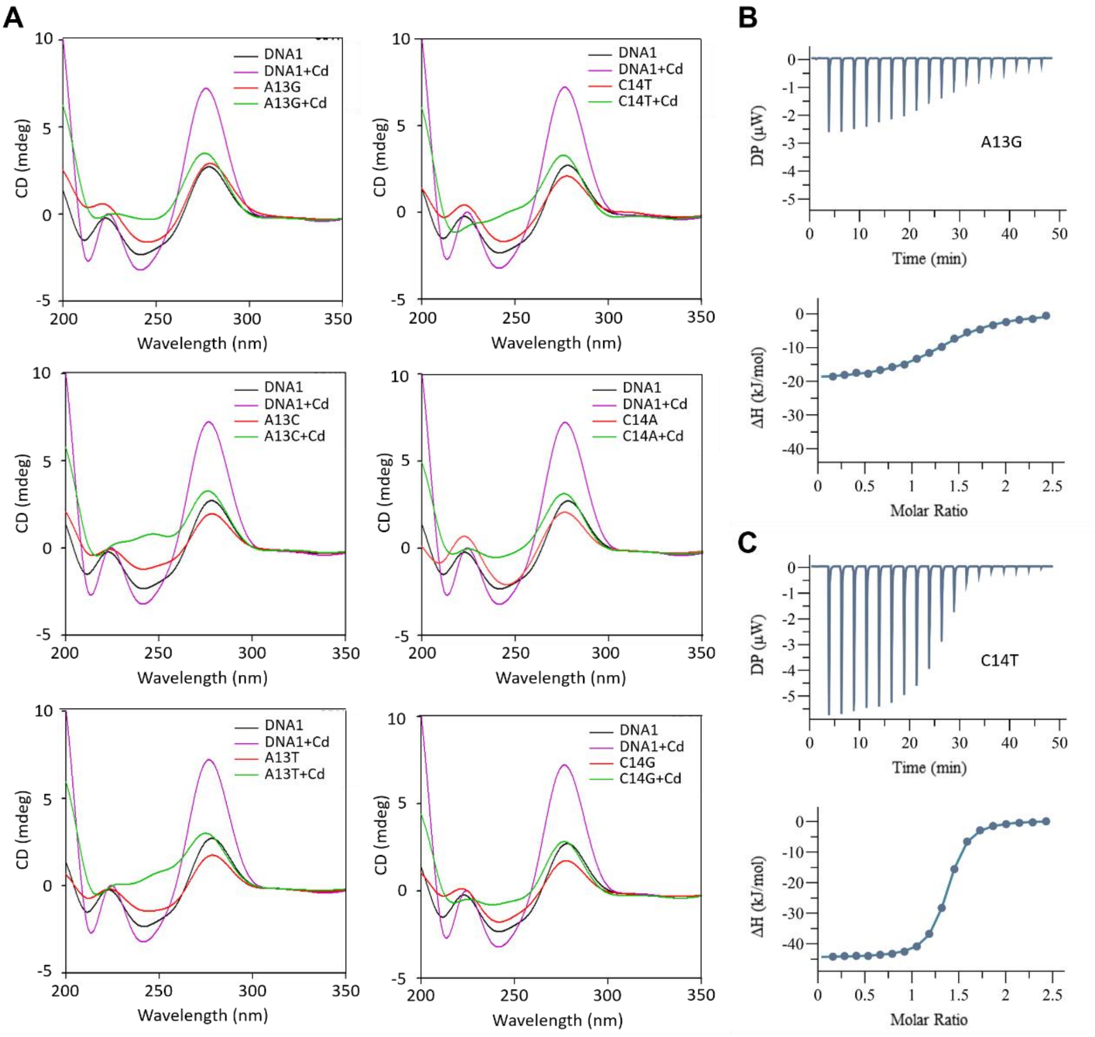

Supplementary Figure S11. Supplementary Figure S8. (**A**) Comparison of the CD spectra of the native DNA1 and variants with either A13 or C14 mutated. (**B-C**) ITC analysis showing Cd^{2+} binding by the A13G and C14T variants, respectively.

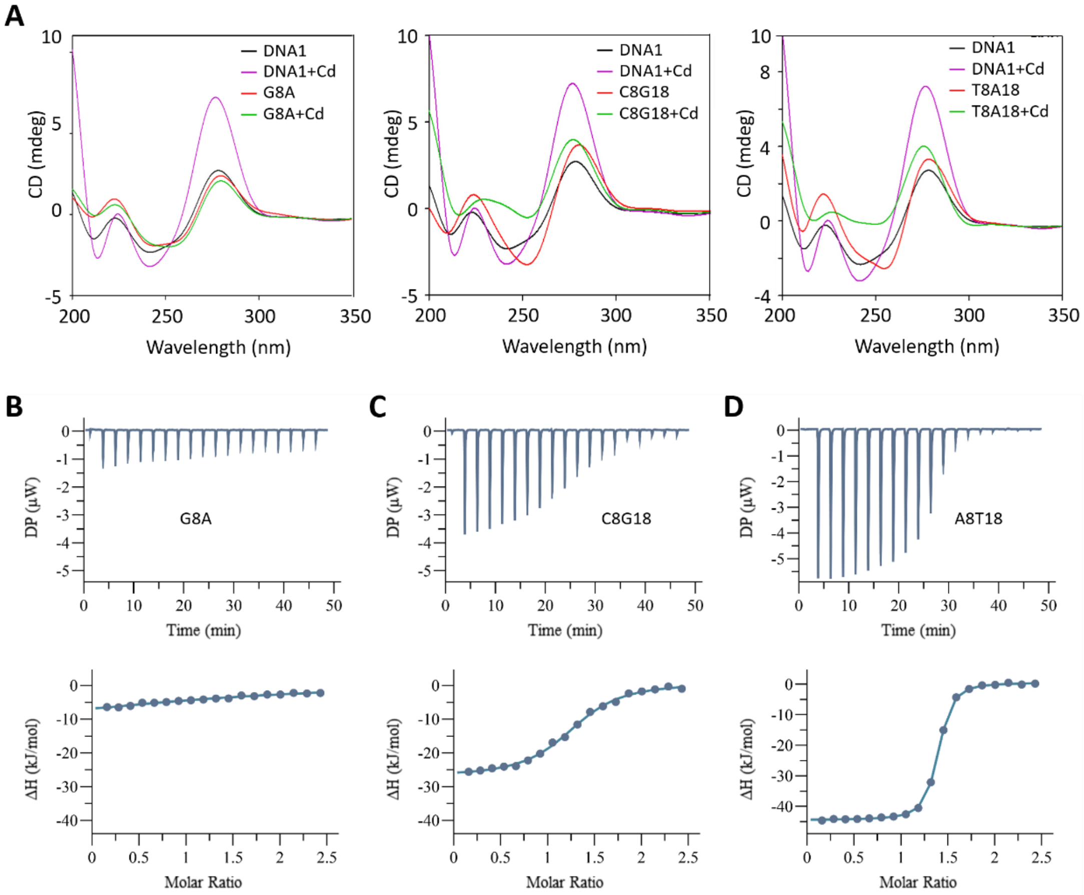

Supplementary Figure S12. (**A**) Comparison of the CD spectra of the native DNA1, the G8A, C8G18 and T8A18 variants. (**B-D**) ITC analysis showing Cd^{2+} binding by the G8A, C8G18 and A8T18 variants, respectively.

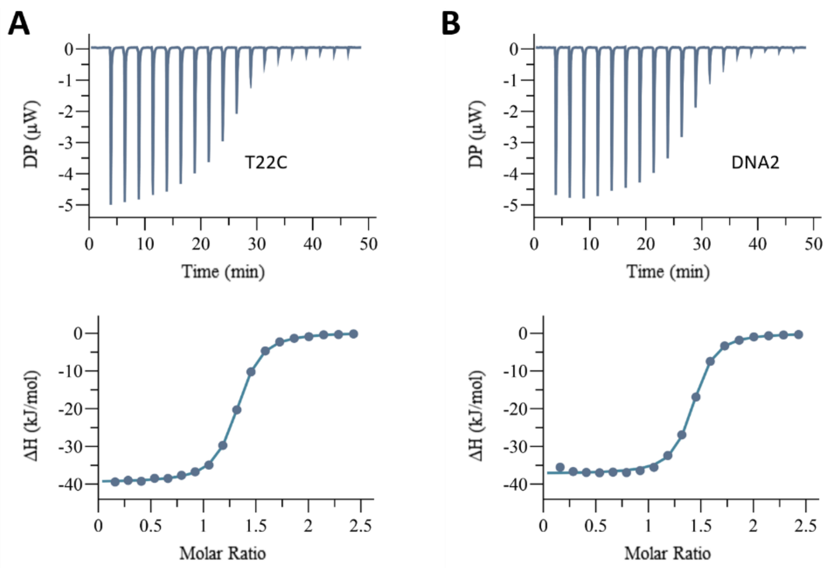

Supplementary Figure S13. ITC analysis showing Cd^{2+} binding by (**A**) the T22C variant and (**B**) DNA2, respectively.

Supplementary Table S1: The detailed sequence of DNA1 and variants.

Name	Sequence (5′-3′)
DNA1	GACGACGGGTTCACAGTCCGTTGTC
G8A	GACGACG**A**GTTCACAGTCCGTTGTC
C8G18	GACGACG**C**GTTCACAGT**G**CGTTGTC
A8T18	GACGACG**A**GTTCACAGT**T**CGTTGTC
T8A18	GACGACG**T**GTTCACAGT**A**CGTTGTC
G9A	GACGACGG**A**TTCACAGTCCGTTGTC
G9T	GACGACGG**T**TTCACAGTCCGTTGTC
G9C	GACGACGG**C**TTCACAGTCCGTTGTC
T10D	GACGACGGG**-**TCACAGTCCGTTGTC
T10F	GACGACGGG**F**TCACAGTCCGTTGTC
T10A	GACGACGGG**A**TCACAGTCCGTTGTC
T10G	GACGACGGG**G**TCACAGTCCGTTGTC
T10C	GACGACGGG**C**TCACAGTCCGTTGTC
C11G15	GACGACGGGT**C**CAC**G**GTCCGTTGTC
G11C15	GACGACGGGT**G**CAC**C**GTCCGTTGTC
A11T15	GACGACGGGT**A**CAC**T**GTCCGTTGTC
C12G	GACGACGGGTT**G**ACAGTCCGTTGTC
C12A	GACGACGGGTT**A**ACAGTCCGTTGTC
C12T	GACGACGGGTT**T**ACAGTCCGTTGTC
A13T	GACGACGGGTTC**T**CAGTCCGTTGTC
A13G	GACGACGGGTTC**G**CAGTCCGTTGTC
A13C	GACGACGGGTTC**C**CAGTCCGTTGTC
C14G	GACGACGGGTTCA**G**AGTCCGTTGTC
C14A	GACGACGGGTTCA**A**AGTCCGTTGTC
C14T	GACGACGGGTTCA**T**AGTCCGTTGTC
A15G	GACGACGGGTTCAC**G**GTCCGTTGTC
G16C	GACGACGGGTTCACA**C**TCCGTTGTC
G16A	GACGACGGGTTCACA**A**TCCGTTGTC
G16T	GACGACGGGTTCACA**T**TCCGTTGTC
T17D	GACGACGGGTTCACAG**-**CCGTTGTC
T17F	GACGACGGGTTCACAG**F**CCGTTGTC
T17A	GACGACGGGTTCACAG**A**CCGTTGTC
T17G	GACGACGGGTTCACAG**G**CCGTTGTC
T17C	GACGACGGGTTCACAG**C**CCGTTGTC
T22C	GCCGACGGGTTCACAGTCCGT**C**GGC
DNA2	--CGACGGGTTCACAGTCCGTCG--

(The T10 and T17 nucleotides of DNA1 were substituted by an abasic site in the T10F and T17F variants, respectively)

Supplementary Table S2: Sample compositions and crystallization conditions.

Structure	Sample composition	Crystallization condition
DNA1-Cd^{2+}	1.0 mM DNA1<br>10 mM $CdCl_2$	10% v/v MPD<br>40 mM Sodium cacodylate pH 6.0<br>12 mM Spermine tetra-HCl<br>80 mM KCl<br>20 mM $BaCl_2$
T10A-Cd^{2+}	1.0 mM DNA1<br>10 mM $CdCl_2$	10% v/v MPD<br>40 mM Sodium cacodylate pH 7.0<br>12 mM Spermine tetra-HCl<br>80 mM KCl<br>20 mM $BaCl_2$
C11G15-Cd^{2+}	1.0 mM DNA1<br>10 mM $CdCl_2$	45% v/v MPD<br>40 mM Sodium cacodylate pH 6.0<br>12 mM Spermine tetra-HCl<br>80 mM NaCl<br>20 mM $BaCl_2$
T22C-Cd^{2+}	1.0 mM DNA1<br>10 mM $CdCl_2$	10% v/v MPD<br>40 mM Sodium cacodylate pH 6.0<br>12 mM Spermine tetra-HCl<br>80 mM NaCl<br>20 mM $BaCl_2$
